# MKL-1 suppresses ferroptosis by activating system Xc- and increasing glutathione synthesis

**DOI:** 10.7150/ijbs.80666

**Published:** 2023-08-21

**Authors:** Zhou-Tong Dai, Yong-Lin Wu, Xing-Rui Li, Xing-Hua Liao

**Affiliations:** 1 Department of Gynaecological Oncology, Tongji Hospital, Tongji Medical College, Huazhong University of Science and Technology, Wuhan, China.; 2 National Clinical Research Centre for Obstetrics and Gynaecology, Cancer Biology Research Centre (Key Laboratory of the Ministry of Education), Tongji Hospital, Tongji Medical College, Huazhong University of Science and Technology, Wuhan, China.; 3 Institute of Biology and Medicine, College of Life and Health Science, Wuhan University of Science and Technology, Wuhan, China.; 4 Department of Thyroid and Breast Surgery, Tongji Hospital, Tongji Medical College, Huazhong University of Science and Technology, Wuhan, China.

## Abstract

Chemotherapy is a standard method in traditional treatment for gastric cancer. It is well known that the anti-tumor effects of chemotherapy are achieved mainly through the direct killing of cancer cells via apoptosis. However, chemotherapy often fails due to drug resistance. Therefore, non-apoptotic cell death induction by ferroptosis has recently been proposed as a new therapeutic modality to ablate cancer. In this study, we determined the role of MKL-1 in ferroptosis. *In vitro* and *in vivo* experiments showed that inhibition of MKL-1 expression significantly enhanced cell sensitivity to ferroptosis-inducing agents. It functions by targeting system Xc- to affect the synthesis of GSH in cells. Therefore, we developed an exosome-based therapeutic approach targeting MKL-1, which provides a novel insight into the treatment of gastric cancer.

## 1. Introduction

Gastric cancer is one of the most common malignant tumors in the world. According to the latest data collected by GLOBOL CANCER, gastric cancer is the fifth most common cancer type and the third most fatal cancer type 1. Although the incidence of gastric cancer has declined globally over the past 50 years due to the prevention and treatment of Helicobacter pylori infection and improved dietary habits, mortality remains high among all cancers 2. For patients with early gastric cancer, surgical resection combined with chemotherapy has been shown to significantly improve the prognosis of some patients with gastric cancer 3. Unfortunately, many patients with gastric cancer are already in the middle-advanced stage or have metastasized when they are diagnosed, and cannot be treated by traditional surgery. Traditional apoptosis-based chemotherapy, targeted drugs or immunotherapy, moderately improve the prognosis of gastric cancer patients 4. The above retrospective analysis found that chemotherapy plays an important role in the treatment of gastric cancer. However, due to the existence of drug resistance mechanism of tumor cells, the efficacy of chemotherapeutic drugs will also be affected by drug resistance during the treatment of gastric cancer patients 5. Most studies have shown that chemotherapeutic drugs kill tumor cells mainly by inducing apoptosis 6. Therefore, ferroptosis is a novel non-apoptotic form of cell death induced by iron-dependent lipid peroxidation. Ferroptosis-based therapy holds promise as a new strategy for antitumor and improving drug resistance of tumor cells.

Ferroptosis is a form of programmed death distinct from apoptosis. In the process of apoptosis, Caspase is activated. At the same time, the cell membrane was intact. During ferroptosis, the accumulation of reactive oxygen species (ROS) disrupts cell membrane integrity 7. It is also very different from cellular necrosis. Although the integrity of the cell membrane is lost during ferroptosis, it does not cause cell swelling 8. A growing number of studies have demonstrated that dysregulation of ferroptosis is highly correlated with human tumors 9. Therefore, the development of therapeutic methods for tumor cell ferroptosis has great application prospects. From the current research progress, multiple mechanisms are involved in the regulation of ferroptosis. According to its definition, it is mainly divided into three main pathways, including iron metabolism pathway, lipid peroxide production pathway and lipid peroxide decomposition pathway 10. However, insufficient progress has been made in the use of ferroptosis for the treatment of tumors.

MKL-1 was initially identified as part of the RBM15-MKL-1 fusion protein, produced in conjunction with the translocated chromosome 22, which is implicated in acute megakaryocytic leukemia in infants and children 11. Typically, MKL-1 resides in the cytoplasm, where it binds to G-actin. However, upon cellular stimulation, the RhoA pathway is activated, leading to the polymerization of G-actin into filamentous actin, F-actin. This process releases MKL-1 from G-actin, allowing it to be transported into the nucleus. Within the nucleus, MKL-1 forms a complex with SRF, initiating the transcription of genes primarily by recognizing the CArG box within the promoter region of certain genes 12. As research has progressed, it has been discovered that MKL-1 plays a role in the regulation of tumor cell proliferation, migration, and apoptosis 13. Notably, a recent study demonstrated that oxidative stress amplifies the mesenchymal transition of human microvascular endothelial cells HMEC-1 via the MKL-1/RhoA/TGF-β pathway 14. Given that ferroptosis is intimately linked with oxidative stress-related diseases 15, these findings suggest a potential role for MKL-1 in the regulation of ferroptosis.

Therefore, in this present study, ferroptosis, a novel form of programmed death based on iron and lipid peroxidation dependence, which we take as an entry point. To clarify the regulation of MKL-1 on the occurrence of ferroptosis in gastric cancer cells and the effect of its expression on ferroptosis inducers. On this basis, the upstream and downstream molecular mechanisms of MKL-1 regulating ferroptosis were further elucidated. And anti-tumor strategies based on ferroptosis therapy targeting MKL-1 were developed for gastric cancer. The results presented a theoretical and experimental basis for the development of novel antitumor drugs.

## 2. Materials and Methods

### 2.1 Cell culture

The gastric cell lines GES-1, MGC-803, MKN-45, SGC-7901, HGC-27, AGS and embryonic kidney cell line HEK-293T were obtained from the Cell Storage Center of Wuhan University (Wuhan, China). Human umbilical cord-derived adipose-derived mesenchymal stem cells (hUC-MSCs) were derived from the Shanghai Cell Bank of the Chinese Academy of Sciences (Shanghai, China). Cells were cultured in the following media: GES-1 and HEK-293T in DMEM supplemented with 10% FBS (Gibco, USA). MGC-803, MKN-45, SGC-7901 and HGC-27 in RPMI-1640 with 10% FBS. AGS cells were cultured in DMEM/F12 with 10% FBS, hUC-MSCs were cultured in Human umbilical cord blood mesenchymal stem cell complete medium (Pricella, China), supplemented with 1% penicillin/streptomycin (Meilunbio, China). Cell lines were tested and verified to be mycoplasma negative using MycoAlert™ PLUS (Lonza, Switzerland). All cells were incubated at 37°C in a humidified atmosphere of 5% CO^2^.

### 2.2 Clinical pathological tissue collection

From 2019 to 2021, clinical pathology samples were obtained from Tongji Hospital. According to the Declaration of Helsinki, the collection of clinicopathological samples has been approved by the Ethics Committee of Tongji Hospital. Samples were plunged into liquid nitrogen immediately after surgery. Subsequently, the paraffin embedding of the samples was entrusted to Wuhan Servicebio Technology Co., Ltd.

### 2.3 Plasmid constructions

The plasmid vector pCDH-CMV, pLKO.1-puro, pGL3-Promoter, pRL-CMV, VSVG and GAG-POL were obtained from Addgene (Addgene, USA). The coding sequence of MKL-1 was inserted into the plasmid pCDH-CMV and used to construct the stable overexpression plasmid pCDH-MKL-1 of MKL-1. Plasmid pLKO.1-puro was used to construct MKL-1 knockdown plasmids pLKO.1-sh-MKL-1-1, pLKO.1-sh-MKL-1-2 and pLKO.1-sh-MKL-1 -3. Plasmid pGL3-Promoter was used to construct luciferase reporter plasmids for the SLC3A2 and SLC7A11 promoters. Plasmid pRL-CMV was used as an internal control plasmid for dual-luciferase reporter activity assays. Plasmids VSVG and GAG-POL were used as lentiviral packaging plasmids. The primer sequences and shRNA sequences were entrusted to Shanghai Sangon Bioengineering Co., Ltd. (Sangong, China) to synthesize.

### 2.4 Lentivirus transduction

Overexpression, knockdown plasmids and package plasmids were transfected into HEK-293T cells using the transfection reagent Lipofectamine 2000 (Invitrogen, USA). The virus-containing supernatants were harvested and filtered through the 0.45 μm filter after 48 h. Cells were plated in 6-well plates at 30-40% confluency per well and transfected with collected lentivirus. The transfected cells were selected by puromycin for two weeks.

### 2.5 Western Blot

Cells were harvested and lysed with RIPA lysis buffer (with PMSF added, Meilunbio, China). The tissue samples were ground with a high-speed tissue grinder (Servicebio, China) lysed with RIPA lysis buffer. After lysis, the samples were disrupted with an ultrasonic cell disruptor, and the supernatant was collected by centrifugation for BCA (Meilunbio, China) protein quantification. The PAGE Gel Rapid Preparation Kit (Yeasen, China) was used to prepare SDS-PAGE gels. The extracted proteins were electrophoresed in SDS-PAGE gels and transferred to polyvinylidene difluoride membranes (PVDF, Millipore, USA). The Blocking solution (Servicebio, China) was used to block the PVDF membrane. Subsequently, the PVDF membrane was immersed in the primary antibody and incubated overnight at 4°C. The next day, a secondary antibody was added for 2 hours at 37°C. After incubation, the membrane was washed with TBST and placed in ECLMeilunbio® Fector's Hypersensitive ECL Luminescent Solution (Meilunbio, China) for 5-10 s. The ChemiDoc XRS gel imaging system was used to collect images. The antibodies used in the Western Blot assay were: Anti-GAPDH (A19056, ABclonal, China), Anti-MKL-1 (77098, CST, USA), Anti-HRP Goat Anti-Rabbit IgG (H+L) (AS014, ABclonal, China), Anti-γ-GCS CST (30068, CST, USA), Anti-GSS (A14535, ABclonal, China), Anti-SLC3A2 (A3658, ABclonal, China), Anti-SLC7A11 (A15604, ABclonal, China), Anti-CD9 (A19027, ABclonal, China), Anti-CD63 (EPR5702, Abcam, USA), Anti-Calnexin (A4846, ABclonal, China).

### 2.6 qRT-PCR assay

QIAGEN RNeasy Mini Kit (QIAGEN, Germany) was used to extract total RNA. The HiScript III 1st Strand cDNA Synthesis Kit (Vazyme, China) was used to reverse-transcribe cDNA. Taq Pro Universal SYBR qPCR Master Mix (Vazyme) was used to perform quantitative real-time RT-PCR analysis, with three replicate wells per sample. The Bio-Rad C1000 Touch PCR instrument (Bio-Rad, USA) was used to perform the qRT-PCR assay. The primer sequence information used in the qRT-PCR assay, GAPDH, F, CTCACCGGATGCACCAATGTT, R, CGCGTTGCTCACAATGTTCAT; MKL-1, F, GGCCAGGACCGAGGACTATT, R, CCACAATGATAGCCTCCTTCAG; FLC, F, CAGCCTGGTCAATTTGTACCT, R, GCCAATTCGCGGAAGAAGTG; FHC, F, CGAGGTGGCCGAATCTTCC, R, GTTTGTGCAGTTCCAGTAGTGA; NCOA4, F, GCTCAGCAGCTCTACTCGTTA, R, GGCACACAGAGACTTGATTGG; ACSL4, F, ACTGGCCGACCTAAGGGAG, R, GCCAAAGGCAAGTAGCCAATA; LPCAT3, F, TTACCTTCAAACCTGGTGCATT, R, CGTGAGCCACAGGATTTCC; ALOX15, F, CCGACCTCGCTATCAAAGACT, R, CACCAGAAAATCCGGTTGAAGT; FSP1, F, GATGAGCAACTTGGACAGCAA, R, CTGGGCTGCTTATCTGGGAAG; DHODH, F, GGAAACCCTAGACCCAGAGTC, R, ACCACTGAAAGCCCGTGAC; GCH1, F, GTGAGCATCACTTGGTTCCAT, R, GTAAGGCGCTCCTGAACTTGT; GPX4, F, GAGGCAAGACCGAAGTAAACTAC, R, CCGAACTGGTTACACGGGAA; γ-GCS, F, GGAGACCAGAGTATGGGAGTT, R, CCGGCGTTTTCGCATGTTG; GSS, F, GGAACATCCATGTGATCCGAC, R, GCCATCCCGGAAGTAAACCA; SLC3A2, F, GTGCTGGGTCCAATTCACAAG, R, CACCCCGGTAGTTGGGAGTA; SLC7A11, F, GGTCCATTACCAGCTTTTGTACG, R, AATGTAGCGTCCAAATGCCAG.

### 2.7 Cell viability assay

Briefly, 2×10^3^ cells were plated in each well of a 96-well plate, and each group was set up with six parallel sub-wells. Then, 10 µL of Cell Counting Kit-8 solution (CCK-8, Vazyme, China) was added to a 96-well plate. Next, the absorbance of the samples at OD 450 nm was measured on the Thermo Varioskan LUX multifunctional microplate reader (Thermo, USA). Calculation formula of cell viability = (experimental group-blank group)/(control group-blank group)×100%.

### 2.8 Transmission electron microscopy (TEM) assay

The electron microscope fixative was added to samples for fixation. Subsequently, the samples were entrusted to Wuhan Servicebio Technology Co., Ltd. (Servicebio, China) for the preparation of ultrathin sections and staining. Samples were negatively stained with 3% phosphotungstic acid solution at room temperature for 5 min. The TEM HITACHI HT7700 (HITACHI, Japan) was used to observe the samples. The accelerating voltage was set at 80KV.

### 2.9 Lipid ROS assay

The C11 BODIPY 581/591 probe (Glpbio, USA) was used to evaluate the lipid ROS production of samples according to the manufacturer's instructions. The cells were digested with trypsin digestion solution without EDTA or phenol red and were collected into EP tubes. The tissue samples were ground by a high-speed tissue grinder, and then filtered through a 40µm cell strainer to obtain a cell suspension. The Lipid ROS detection working solution was added to the samples and incubated at 37°C for 20 minutes. After centrifugation, PBS was used to resuspend the cells. The BD FACSMelody flow cytometer (BD, USA) was used to analyze the samples for lipid ROS production.

### 2.10 GSH assay

The GSH detection kit (Beyotime, China) was used to evaluate the relative GSH level of samples according to the manufacturer's instructions. The absorbance of the samples at OD 412 nm was measured on the Thermo Varioskan LUX multifunctional microplate reader. The standard curve was drawn against the standard samples, and the relative GSH level in the samples was calculated by the protein concentration of the samples.

### 2.11 Malonyldialdehyde (MDA) assay

The MDA detection kit (Beyotime, China) was used to evaluate the relative MDA content of samples according to the manufacturer's instructions. The absorbance of the samples at OD 532 nm was measured on the Thermo Varioskan LUX multifunctional microplate reader. The standard curve was drawn against the standard samples, and the relative MDA level in the samples was calculated by the protein concentration of the samples.

### 2.12 Iron assay

The Iron Assay Kit (Abcam, UK) was used to evaluate the relative Fe^2+^ content of samples according to the manufacturer's instructions. The absorbance of the samples at OD 593 nm was measured on the Thermo Varioskan LUX multifunctional microplate reader. The standard curve was drawn against the standard samples, and the relative Fe^2+^ level in the samples was calculated by the protein concentration of the samples.

### 2.13 Glutamate, Glycine and Cysteine assay

The glutamate glycine and cysteine measurement kit (Nanjing Jiancheng, China) was used to evaluate the relative glutamate, glycine and cysteine content of samples according to the manufacturer's instructions. The absorbance of the samples was measured on the Thermo Varioskan LUX multifunctional microplate reader. The standard curve was drawn against the standard samples, and the relative glutamate, glycine, and cysteine level in the samples was calculated by the protein concentration of the samples.

### 2.14 NADPH assay

The NADPH oxidase activity reagent box (Nanjing Jiancheng, China) was used to evaluate the relative NADPH content of samples according to the manufacturer's instructions. The absorbance of the samples was measured on the Thermo Varioskan LUX multifunctional microplate reader. The standard curve was drawn against the standard samples, and the relative glutamate, glycine and cysteine level in the samples was calculated by the protein concentration of the samples.

### 2.15 Small RNA-sequencing

Small RNA-seq was entrusted to Shanghai Sangon Bioengineering Co., Ltd.

### 2.16 Dual-luciferase reporter gene assay

The Dual-Luciferase Reporter Assay System (Promega, USA) was used to evaluate the relative luciferase activity of samples according to the manufacturer's instructions. Briefly, the dual-luciferase reporter plasmid was transfected into the cell line. After 48 hours, the absorbance of the samples was measured on the Thermo Varioskan LUX multifunctional microplate reader for luciferase activity, and the Renilla luciferase was used as a control. Meanwhile, two additional sub-wells were set up in each group.

### 2.17 RNA immunoprecipitation (RIP) assay

RIP experiments were performed using a RIP kit (Gisa Biotechnology, China) following the manufacturer's instructions. Anti-AGO2 and anti-IgG were used for immunoprecipitation, and qRT-PCR was used to analyze target RNA. The antibody information used in RIP assay, Anti-AGO2 (A19709, ABclonal, China), Anti-IgG (AC011, ABclonal, China).

### 2.18 Extraction and characterization of exosomes

The VEX Exosome Isolation Reagent (Vazyme, China) was used to extract exosomes from the cell culture medium according to the manufacturer's instructions. The TEM and Nanosight NS 300 (Nanosight, UK) were used to analyze the morphological features and size of exosomes and size.

### 2.19 Induction of differentiation of mesenchymal stem cells (MSCs) assay

MSC osteogenic and adipogenic induction differentiation assays were used to identify the differentiation potential of MSCs. The culture medium of the adherent hUC-MSCs was changed to human umbilical cord mesenchymal stem cell osteogenic differentiation medium (Pricella, China) or human umbilical cord mesenchymal stem cell adipogenic differentiation medium (Pricella, China). The fresh medium was changed every 2-3 days. Three weeks after induction of differentiation, 4% paraformaldehyde was used to fix hUC-MSCs. Alizarin Red staining solution and Oil Red O staining solution were used to stain it. The inverted microscope was used to obtain images.

### 2.20 Exosome transformation protocol

HUC MSCs were used as parental cells to prepare therapeutic exosomes that overexpressed miR-149-5p and encapsulated MKL-1 siRNA by lentivirus packaging and electroporation. The electroporation protocol was set at 0.15 KV/100 µV. The electroporation method and experimental conditions were referred to in this document 16.

### 2.21 Exosome uptake assay

PKH26 kit (Sigma, USA) was used to label exosomes. The PKH26 working solution was added to the exosomes resuspended in Diluent C according to the instructions. After incubation, ultracentrifugation (100 000 rpm) was used to remove the free PKH26 working solution. Subsequently, exosomes resuspended in PBS were added to the cells for co-culture. After the incubation period, all medium was discarded. 4% paraformaldehyde was used for fixation. DAPI staining solution was used to stain nuclei. The OLYMPUS FV3000 Confocal Microscope was used for fluorescence detection.

### 2.24 EdU assay

The EdU assay kit used was BeyoClick™ EdU Cell Proliferation Detection Kit (Beyotime, China). Briefly, the logarithmically growing target cells were collected and seeded into 35mm glass bottom dishes (Biosharp, China). After the termination of the incubation, the EdU working solution was prepared and labelled as described in the instructions. The confocal microscope was used to photograph images, and Image J was used to count EdU-positive cells.

### 2.25 Colony formation assay

Logarithmically growing cells of interest were collected and seeded into 6-well plates and cultured in an incubator for 14 days. The 4% paraformaldehyde, 0.1% crystal violet staining solution and PBS were used to fix, stain, and wash. Subsequently, the 6-well plate was placed in a fume hood to dry. Colonies larger than 50 cells were counted under a microscope.

### 2.26 Immunohistochemistry (IHC) assay

The samples were placed in the tissue fixative (Meilunbio, China) for more than 24 hours. The pathological tissue samples were dehydrated and transparent, dipped in wax, and embedded in sequence. Paraffin sections of 3 mm samples were prepared on a paraffin microtome. The paraffin sections were sequentially subjected to dewaxing and hydration, antigen retrieval, endogenous peroxidase inactivation, blocking, primary antibody incubation, secondary antibody incubation, DAB color development, hematoxylin staining, hematoxylin reverse blue, and dehydration operations. Added 5-10 µL of neutral resin dropwise (Sinopharm Chemical Reagent, China) to the stained area and mounted the sections. The stained sections of pathological tissue samples were placed under a slide scanner for scanning, and quantitative statistical analysis was performed. The IHC score was calculated by combining the ratio score (percentage of positively stained cells) with the staining intensity score. The scale and staining intensity scores were then multiplied to generate a score for each case. The antibody information used in IHC assay, Anti-MKL-1 (77098, CST, USA) Anti-SLC3A2 (A3658, ABclonal, China), Anti-SLC7A11 (A2413, ABclonal, China).

### 2.27 Nude mice xenograft model

The experimental animals BALB/C nude mice were purchased from Beijing Huafukang Biotechnology Co., Ltd. (Huafukang, China) and raised in the SRF environment on the 3rd floor of the Animal Experiment Center of Wuhan University of Science and Technology. The litter, feed and drinking water of BALB/C nude mice were changed twice a week. This animal experiment on BALB/C nude mice has been reviewed and approved by the Ethics Committee of the School of Life Science and Health of Wuhan University of Science and Technology and the Laboratory Animal Center of Wuhan University of Science and Technology. For subcutaneous inoculation, cells resuspended in PBS at 2×10^7^ cells/1mL (100μL) were injected into four weeks old nude mice. The tumors were measured every three days. The formula was (1/2×length×width×width).

### 2.28 Radiotracer Cell Uptake

Cells were initially cultured in cysteine-depleted medium for three days in 6-well plates at an appropriate density. At the start of the experiment, isotopically labeled cysteine (200 μmol/L, [U-^13^C^6^, U-^15^N^2^] cystine, purchased from MCE, USA) was added to the medium. After an incubation period of 1 hour at 37°C, the medium was removed, and the cells were processed for analysis. The uptake of isotopically labeled cysteine in the cells was measured using liquid chromatography-mass spectrometry (LC-MS).

### 2.29 Data Analysis

Primer design was undertaken using Primer Premier 5 software, whereas Image Lab software was utilized for the analysis of Western Blot results. Analysis of both Western Blot and IHC results was conducted with Image J software, and FCM results were analyzed using FlowJo VX software. R software was employed for processing bioinformatics results. All experimental results are reported as mean ± standard deviation (SD). In statistical evaluations, the Student's t-test was applied when comparisons were made between two groups. For comparisons among multiple groups, one-way ANOVA test was used. Post-hoc analyses were performed using either Dunnett's or Tukey's test to ascertain significant differences between groups. For the measurement of tumor growth over time, statistical significance was determined using two-way ANOVA or multiple t-test. In the figures, statistical significance is denoted as follows: * for *p* < 0.05, ** for *p* < 0.01, and *** for *p* < 0.001.

## 3. Results

### 3.1 MKL-1 was up-regulated in gastric cancer tissues and cell lines

**Figure 1:**
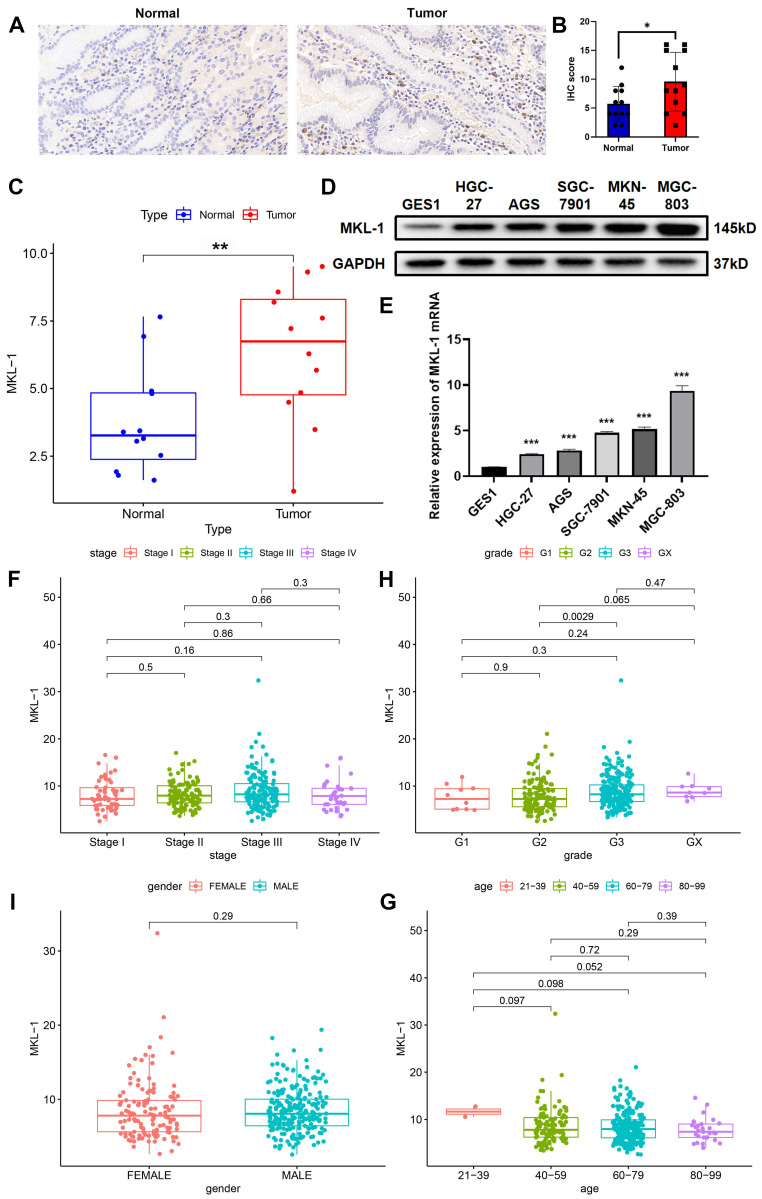
MKL-1 was up-regulated in gastric cancer tissues and cell lines. A. Typical images of IHC staining of tumor tissues and adjacent tissues of gastric cancer patients. B. IHC staining score of MKL-1 (n = 12 patients/group). The paired t-test was used to determine the statistical significance. C. The expression of MKL-1 in tumor tissues and adjacent tissues detected by qRT-PCR (n = 12 patients/group). The paired t-test was used to determine the statistical significance. D. Western blot was used to detect the expression of MKL-1 in cell lines GES1, AGS, MKN-45, HGC-27, SGC-7901 and MGC-803. E. The expression of MKL-1 mRNA in cell lines GES1, AGS, MKN-45, HGC-27, SGC-7901 and MGC-803 was detected by qRT PCR. F-I. Analysis of the correlation between MKL-1 expression in STAD patients in the TCGA database and their corresponding stage, grade, gender, and age. The Western blot and qRT-PCR results represented the average values obtained from three independent experiments. The results shown are Mean ± SD. * *p* < 0.05, ** *p* < 0.01, *** *p* < 0.001.

In previous studies by our group, it has been confirmed that MKL-1 was closely related to the occurrence and development of cancer 17. Furthermore, we downloaded the expression profile data of gastric cancer patients from the TCGA database. It was found that the expression of MKL-1 in gastric cancer was up-regulated, and the overall survival rate of gastric cancer patients in the high-expression group was lower. Therefore, the expression of MKL-1 in gastric cancer tissues and adjacent tissues was detected by qRT-PCR and IHC staining (Figure 1A-C). It was further confirmed that MKL-1 was significantly up-regulated in gastric cancer tumor tissues. At the same time, the same phenomenon was found in different human gastric cancer cells (Figure 1D-E). The relationship between MKL-1 expression and clinicopathological characteristics of gastric cancer patients was further analyzed through the TCGA database. It was found that the expression of MKL-1 was not related to the stage, gender and age of gastric cancer patients (Figure 1F-I). Although there were significant differences between grade 2 and grade 3, there was no significant difference between grade 1 and grade 2 or between grade 1 and grade 3. The above results showed that the expression of MKL-1 was up-regulated in gastric cancer patients, and its increase had no significant correlation with their clinical and pathological characteristics. This data provided a theoretical basis for using MKL-1 as a potential gastric cancer marker.

### 3.2 Knockdown of MKL1 enhanced Erastin-induced ferroptosis in gastric cancer

**Figure 2:**
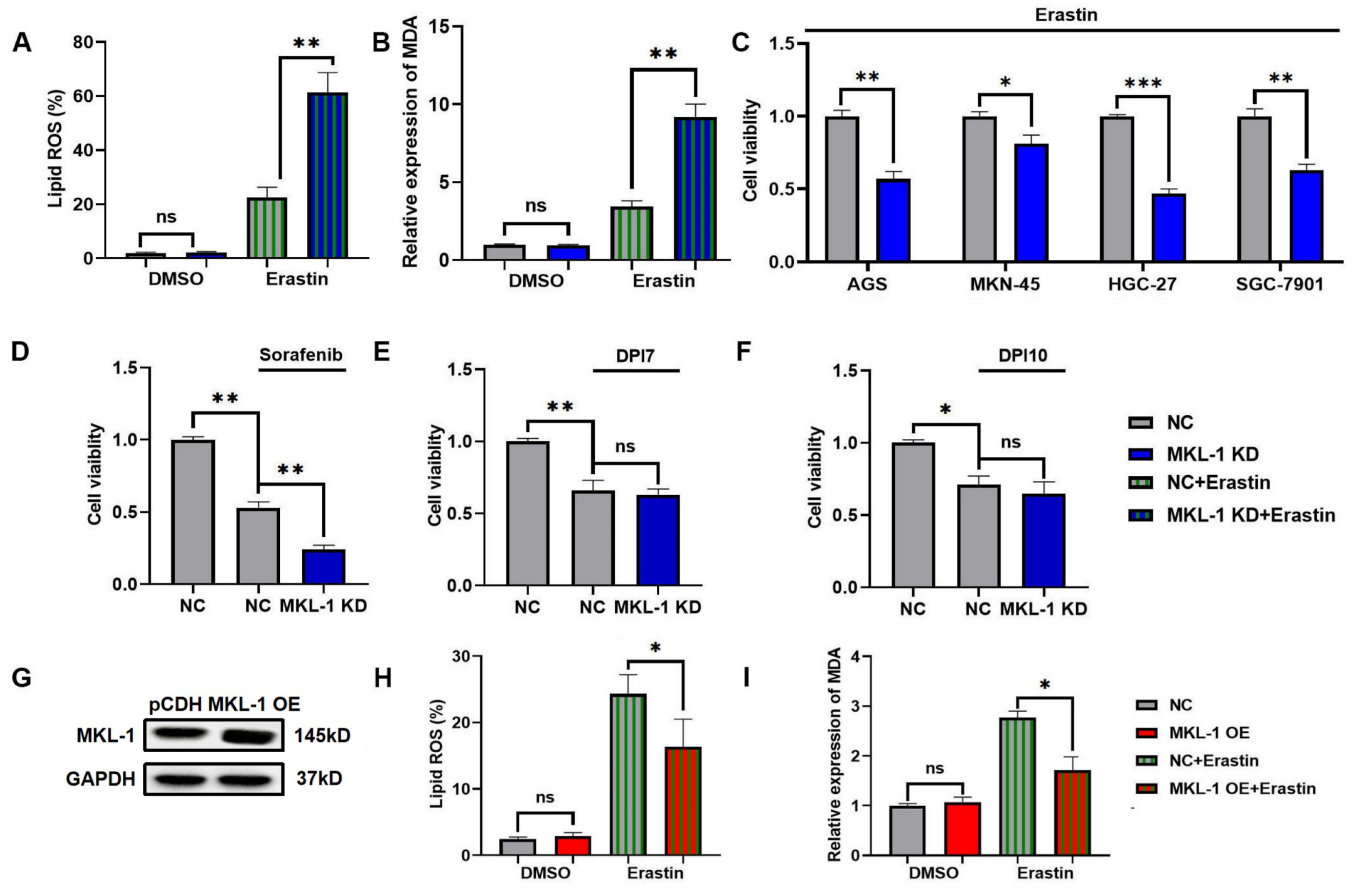
Knockdown of MKL1 enhanced Erastin-induced ferroptosis in gastric cancer. A. After the knockdown of MKL-1 expression, the Lipid ROS level of cells was detected by C11 BODIPY 581/591 probe and FCM. B. After the knockdown of MKL-1 expression, the level of the membrane lipid peroxidation product MDA was detected by the MDA lipid oxidation level detection kit. C. CCK-8 assay was used to detect cell viability of human gastric cancer cell lines after the addition of ferroptosis inducer Erastin. D-F. CCK-8 assay was used to detect cell viability of human gastric cancer cell lines after the addition of different ferroptosis inducers Sorafenib, DPI7 or DPI10. G. The protein expression of MKL-1 in cells was detected by Western Blot. H-I. After overexpressed MKL-1 expression in HGC-27, the Lipid ROS and MDA assays. The t-test was used to determine the statistical significance for Lipid ROS, MDA and CCK-8. The Western blot, Lipid ROS assay, MDA assay and CCK-8 assay results represented the average values obtained from three independent experiments. The results shown are Mean ± SD. * *p* < 0.05, ** *p* < 0.01, *** *p* < 0.001, ns = *p* > 0.05.

To study the role of MKL-1 in the regulation of cellular ferroptosis, we induced ferroptosis using Erastin, a common ferroptosis promoter 15. We applied different concentrations of Erastin to adherent MGC-803 and HGC-27 cell lines. The CCK-8 assay results demonstrated that the cell viability of MGC-803 and HGC-27 significantly decreased upon Erastin application (Figure S1A-B). Additionally, Erastin exerted a more potent inhibitory effect on the growth of the HGC-27 cell line, which exhibits lower MKL-1 expression, compared to MGC-803. To further confirm Erastin's role in enhancing the ferroptosis process in MGC-803 and HGC-27 cell lines, we used Ferrostatin-1 (Fer-1), a specific ferroptosis inhibitor, to counteract the effects of Erastin 15. The accumulation of lipid ROS in cells was detected using a C11 BODIPY 581/591 probe, and membrane lipid peroxidation products were identified using an MDA assay kit. We observed that Fer-1 effectively reversed the lipid ROS accumulation and MDA production induced by Erastin (Figure S1C-D). Subsequently, we analyzed the morphology of the mitochondria in cells, another characteristic of ferroptosis, using transmission electron microscopy (TEM) (Figure S1E). Building upon these observations, we employed lentiviral packaging technology to alter MKL-1 expression in a human gastric cancer model under ferroptosis stress (Figure S2A-C). The results indicated that the effect of Erastin in inducing ferroptosis in gastric cancer cells was significantly amplified in MKL-1 KD cells (Figure 2A-B). To further solidify our findings, we knocked down the expression of MKL-1 in MKN-45, SGC-7901, HGC-27, and AGS human gastric cancer cell lines (Figure S2D). We found that Erastin significantly reduced cell viability and that MKL-1 knockdown further amplified the anti-tumor effect of Erastin (Figure 2C). This suggests that the regulation of ferroptosis by MKL-1 is not restricted to specific gastric cancer cell lines.

We also investigated the ferroptosis status of gastric cancer cells under the influence of different ferroptosis inducers. Interestingly, the same phenomenon observed in Erastin-induced MGC-803 was also noted after sorafenib treatment. However, the knockdown of MKL-1 expression did not significantly impact cell viability following treatment with either DPI7 or DPI10 (Figure 2D-F). Both Erastin and Sorafenib induce ferroptosis by inhibiting system Xc-, while DPI7 and DPI10 directly bind to GPX4 covalently, causing its inactivation and thus inducing ferroptosis. Concurrently, overexpression of MKL-1 significantly mitigated the ferritin deposition event induced by Erastin (Figure 2G-I). Therefore, we hypothesize that MKL-1 may influence cellular ferroptosis by participating in the regulation of system Xc-.

### 3.3 Suppression of MKL-1 enhanced the anti-tumor effect of ferroptosis inducers *in vivo*

**Figure 3:**
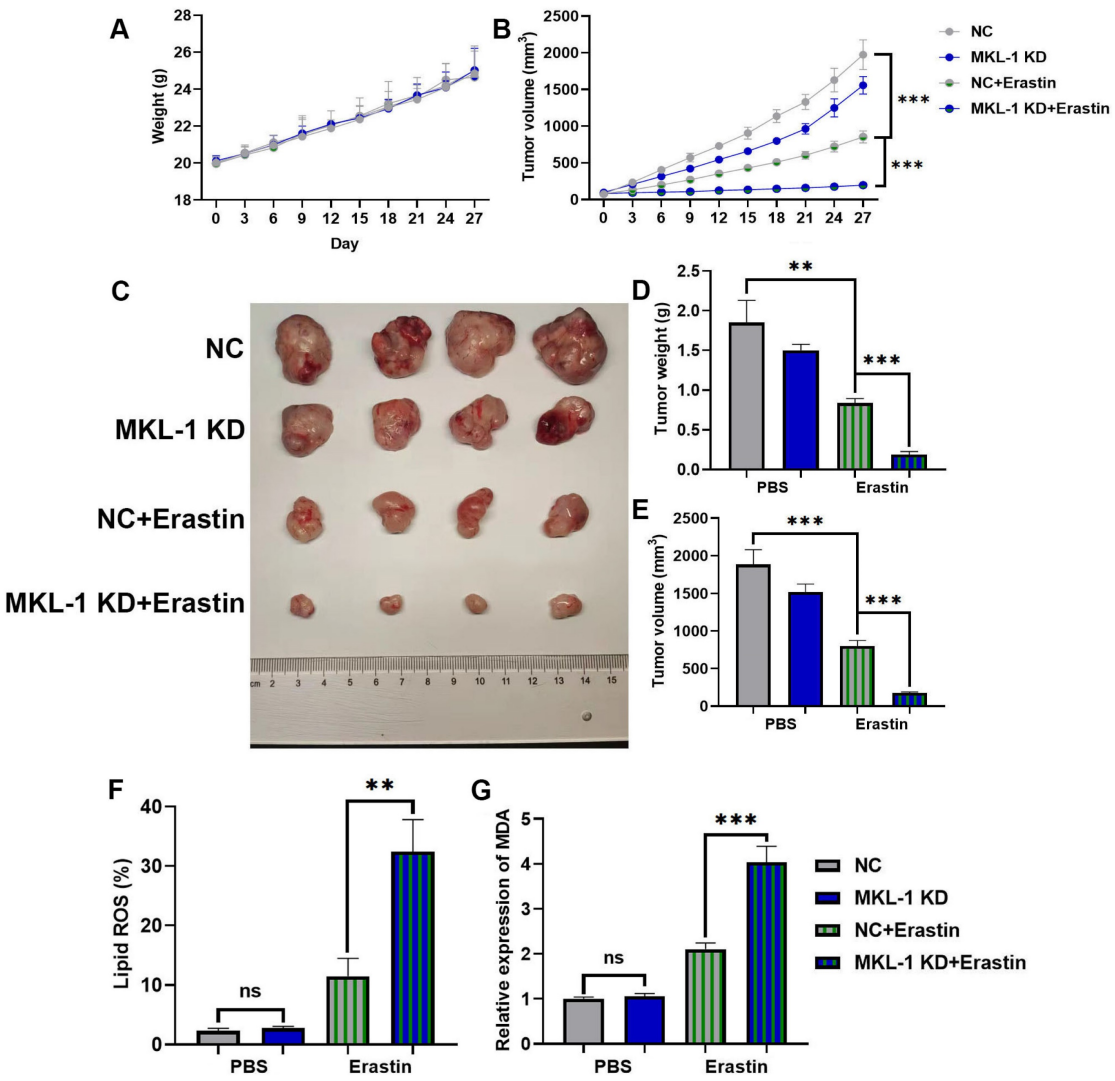
Suppression of MKL-1 enhanced the anti-tumor effect of ferroptosis inducers *in vivo*. A. The growth curve of the body weight of nude mice after Erastin treatment (n = 4-8 tumors /group). B. The growth curve of the subcutaneous tumor volume of nude mice measured *in vitro* after Erastin treatment. Two-way ANOVA (Sidak's multiple comparisons test) was used to determine the statistical significance for body weight and tumor volume. C. Typical images of the subcutaneous tumor tissue of nude mice after Erastin treatment. D. The mass of subcutaneous tumor tissue in nude mice after Erastin treatment. E. The volume of subcutaneous tumor tissue in nude mice after Erastin treatment. F. The level of Lipid ROS in subcutaneous tumor tissue of nude mice was detected by C11 BODIPY 581/591 probe and FCM. G. The level of the membrane lipid peroxidation product MDA in subcutaneous tumor tissue of nude mice was detected by the MDA lipid oxidation level detection kit. The t-test was used to determine the statistical significance for Lipid ROS, MDA, tumor mass and volume. The t-test was used to determine the statistical significance for both groups of analysis. The Western blot, Lipid ROS assay, MDA assay and CCK-8 assay results represented the average values obtained from three independent experiments. The results shown are Mean ± SD. * *p* < 0.05, ** *p* < 0.01, *** *p* < 0.001, ns = *p* > 0.05.

All of the above experimental results showed that the knockdown of MKL-1 expression at the cellular level enhanced the anti-tumor effect of Erastin. Next, its function was explored *in vivo* by a nude mouse xenograft tumor model. MGC-803 cells and MKL-1 knockdown MGC-803 cells were inoculated into the hind limbs and dorsal subcutaneous areas of nude mice, respectively. After the 7th day of inoculation, tumor volumes of 70-100 mm^3^ were selected for follow-up experiments. The experimental group was injected with a total volume of 100 µl of 20 mg/kg Erastin intratumorally every two days, and the control group was injected with an equal volume of PBS. Body weights of nude mice did not show significant differences between all groups during the treatment (Figure 3A). However, after treatment with Erastin, tumor growth was slower in nude mice in the Erastin-treated group compared to the untreated group (Figure 3B). Meanwhile, the MKL-1 knockdown group significantly enhanced the therapeutic effect of Erastin (Figure 3C-E). Moreover, subcutaneous tumor tissues were isolated. We found that the lipid ROS and MDA were also significantly increased in the knockdown MKL-1 expression group after treatment (Figure 3F-G). Thus, our data suggest that the knockdown of MKL-1 expression enhances the anti-tumor effect of Erastin *in vivo*. It showed that MKL-1 expression plays a key role in the process of ferroptosis *in vivo*.

### 3.4 MKL-1 regulated GSH synthesis via System Xc-

**Figure 4:**
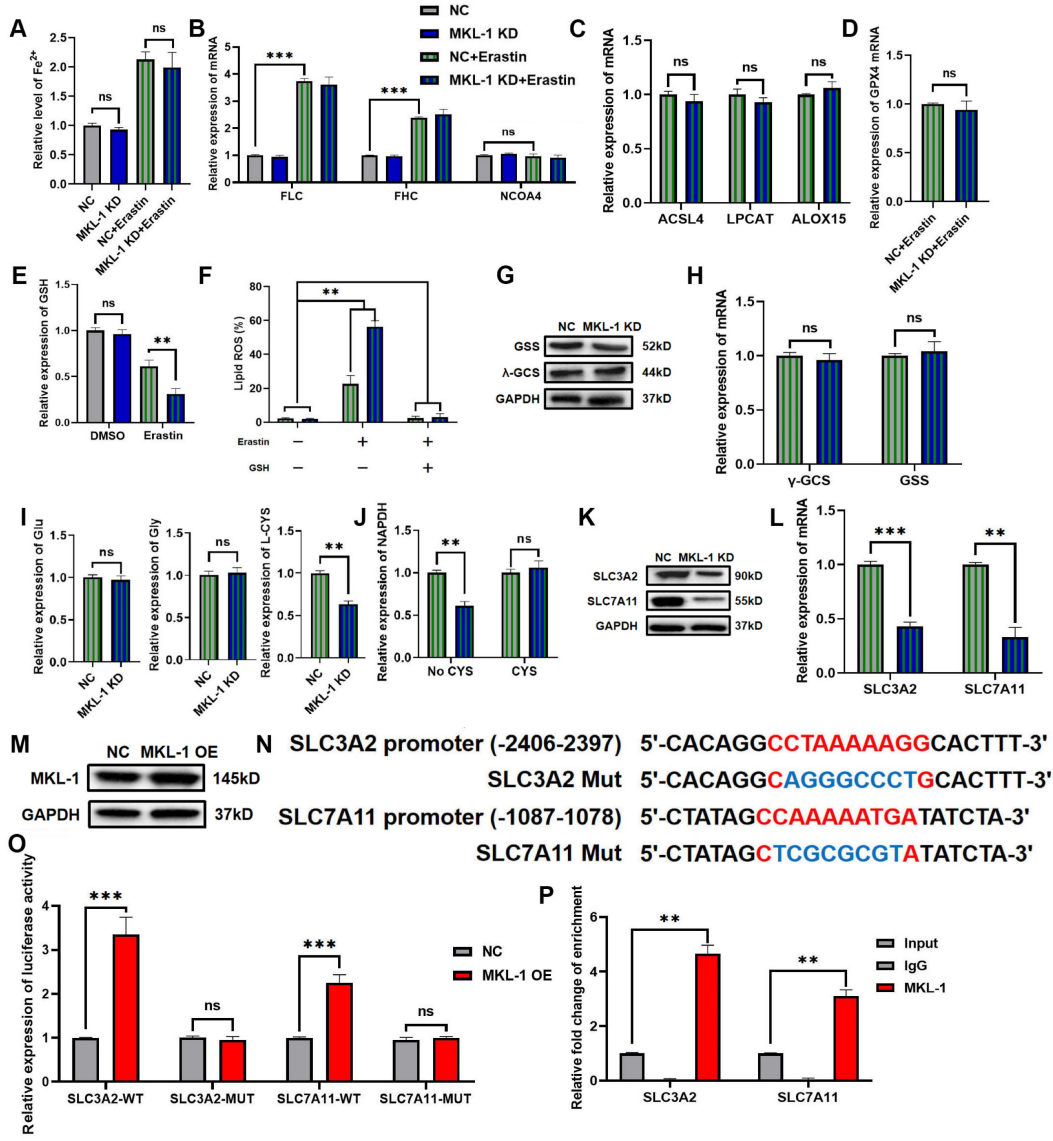
MKL-1 regulated GSH synthesis via System Xc-. A. The level of Fe^2+^ ion in cells was detected by ferrous ion detection kit. B. The mRNA expression of FLC, FHC and NCOA4 were detected by qRT-PCR. C. The mRNA expression of ACSL4, LPCAT and ALOX-15 were detected by qRT-PCR. D. The mRNA expression of GPX4 was detected by qRT-PCR. E. The level of GSH in cells was detected by the GSH detection kit. F. The level of Lipid ROS in cells was detected by C11 BODIPY 581/591 probe and FCM. G. The protein expression of γ-GCS and GSS in cells was detected by Western Blot. H. The mRNA expression of γ-GCS and GSS in cells was detected by qRT-PCR. I. The level of Glu, Gly and L-CYS in cells was detected by the detection kit. J. The level of NADPH in cells was detected by NADPH oxidase activity reagent box. K. The protein expression of SLC3A2 and SLC7A11 in cells was detected by Western Blot. L. The mRNA expression of SLC3A2 and SLC7A11 in cells was detected by qRT-PCR. M. The protein expression of MKL-1 in cells was detected by Western Blot after transfection of pcDNA 3.1-MKL-1 plasmid and pcDNA 3.1 plasmids in cells. N. Schematic diagram of the promoter binding sites of MKL-1 and SLC3A2 or SLC7A11. O. After transfection of different luciferase reporter plasmids, the luciferase activity in the cells was detected by a microplate reader. P. The promoter binding of MKL to SLC3A2 and SLC7A11 was detected by ChIP experiments. The t-test was used to determine the statistical significance for both groups of analysis. The Fe^2+^ ion, Western blot, Lipid ROS assay, GSH assay NADPH assay, Glu, Gly, L-CYS assay, Luciferase assay and ChIP assay results represented the average values obtained from three independent experiments. The results presented were Mean ± SD. * p < 0.05, ** *p* < 0.01, *** *p* < 0.001, ns = *p* > 0.05.

As a new form of iron-dependent programmed cell death, ferroptosis was mainly involved in the regulation of programmed cell death through the following pathways. These pathways were the iron metabolism pathway, lipid peroxide production pathway and lipid peroxide decomposition pathway 10. Meanwhile, MKL-1 formed a complex with SRF and participated in the regulation of gene transcription 18. Therefore, the level of Fe^2+^ in cells was detected by the iron content detection kit, and the mRNA expressions of ferritin light chain (FLC), ferritin heavy chain (FHC) and NCOA4 in cells were detected by qRT-PCR. The Fe^2+^ level in the cells was significantly increased after the addition of the Erastin ferroptosis inducer compared to the non-added group (Figure 4A). However, the Fe^2+^ level in the MKL-1 knockdown group was not significantly different from that in the control group, whether in the Erastin-treated group or the untreated group (Figure 4A). Likewise, qRT-PCR also showed the same results (Figure 4B). The above results showed that MKL-1 does not directly regulate the occurrence of ferroptosis through the iron metabolism pathway.

Another characteristic feature of ferroptosis was an increase in lipid peroxides. ACSL4, LPCAT and ALOX played important roles in the production of lipid peroxides 19. Surprisingly. After the addition of the Erastin ferroptosis inducer, the knockdown of MKL-1 did not affect the mRNA expression of ACSL4, LPCAT and ALOX-15 (Figure 4C). It implied that MKL-1 does not directly regulate the occurrence of ferroptosis through the production of lipid peroxides.

The occurrence of ferroptosis is closely related to the accumulation of lipid peroxides. Therefore, the antioxidant-reduction system was also widely involved in the regulation of ferroptosis 10. GPX4 was a selenozyme that utilizes two GSHs as electron donors to reduce lipid peroxides to the corresponding alcohols and generate GSSG, thereby reducing lipid peroxidation and preventing ferroptosis in cells 20. Therefore, in the ferroptosis stress model established using Erastin ferroptosis inducer, the effect of knocking down the expression of MKL-1 on the expression of GPX4 mRNA was detected by qRT-PCR. Compared with the control group, the expression of GPX4 mRNA did not change in the MKL-1 knockdown group (Figure 4D). Next, the effect of changes in MKL-1 expression in cells on the level of GSH was detected by the GSH detection kit. GSH level was significantly lower in Erastin-treated groups compared to untreated groups. Meanwhile, the knockdown of MKL-1 expression further reduced the level of GSH in cells, from 0.61±0.07 in the control group to 0.31±0.06. This result also verified the results of (Figure 4E) from the side in figure 2D-F. The knockdown of MKL-1 expression did not alter its inhibitory effect after using GPX4 inhibitors, DPI7 and DPI10. However, it affected the effect of system Xc- inhibitors Erastin and Sorafenib (Figure 2D-F). Furthermore, exogenous GSH supplementation also abolished the knockdown effect of MKL-1 expression (Figure 4F). It indicated that the knockdown of MKL-1 expression regulated the occurrence of ferroptosis in ferroptosis stress model by affecting the synthesis of GSH.

GSH in cells is synthesized from glutamate, cysteine, and glycine by γ-GCS enzymes, GSS enzymes, and ATP 21. We found that knocking down the expression of MKL-1 did not affect the levels of γ-GCS enzymes and GSS enzymes *in vitro* (Figure 4G-H). Likewise, the levels of glutamate or glycine in cells were not affected (Figure 4I). However, the knockdown of MKL-1 significantly reduced the cysteine level and the depletion of NADPH in the cells (Figure 4I-J). Because cysteine in cells was mainly imported into cells by the system Xc- 22, it was rapidly converted to cysteine by depleting NADPH. Moreover, findings from our isotopic tracing experiments revealed that overexpression of MKL-1 significantly enhanced the uptake of cystine in cells under the stress of Erastin-induced ferroptosis (Figure S3A-B). Therefore, we hypothesized that the knockdown of the MKL-1 interferes with cysteine uptake pathway in cells.

As we speculated, the knockdown of MKL-1 significantly reduced the expression of two key retrotransporters SLC3A2 and SLC7A11 in system Xc- (Figure 4K-L). Bioinformatics analysis combined with dual luciferase analysis and ChIP also confirmed that MKL-1 promotes their transcription through the direct binding of CArG box binding sites to SLC3A2 and SLC7A11 promoters (Figure 4 M-P, Figure S4B). Our preliminary investigations, as depicted in Figure 2G, evaluated the role of MKL-1 overexpression in attenuating Erastin-induced ferroptosis in gastric cancer cells. Furthering this line of enquiry, we explored whether overexpressing MKL-1 could mediate the synthesis of glutathione (GSH) in cells, thereby resisting the occurrence of ferroptosis. Our results revealed that the overexpression of MKL-1 significantly diminished the decrease in GSH levels induced by Erastin (Figure S4A). This suggests that the modulation of GSH levels through MKL-1 might be an integral mechanism in regulating ferroptosis in response to Erastin. Confirmatory *in vivo* experiments further demonstrated that the overexpression of MKL-1 in gastric cancer cells indeed resisted the onset of Erastin-induced ferroptosis (Figure S4C-F). Overexpression of MKL-1 helped gastric cancer cells synthesize more GSH (Figure S4G). Additionally, we performed correlation analyses that displayed a significant positive correlation between the mRNA expression levels of MKL-1 and SLC3A2, and SLC7A11. This implies a potential relationship between MKL-1 and SLC3A2 or SLC7A11, further elucidating the functional context of MKL-1 in regulating ferroptosis. Taken together, these findings suggest that MKL-1 increases cystine uptake in cells by promoting System Xc-. This increased GSH in cells and decreased ferroptosis in tumor cells.

### 3.5 miR-149-5p triggered ferroptosis in gastric cancer via MKL-1 in the presence of ferroptosis inducers

**Figure 5:**
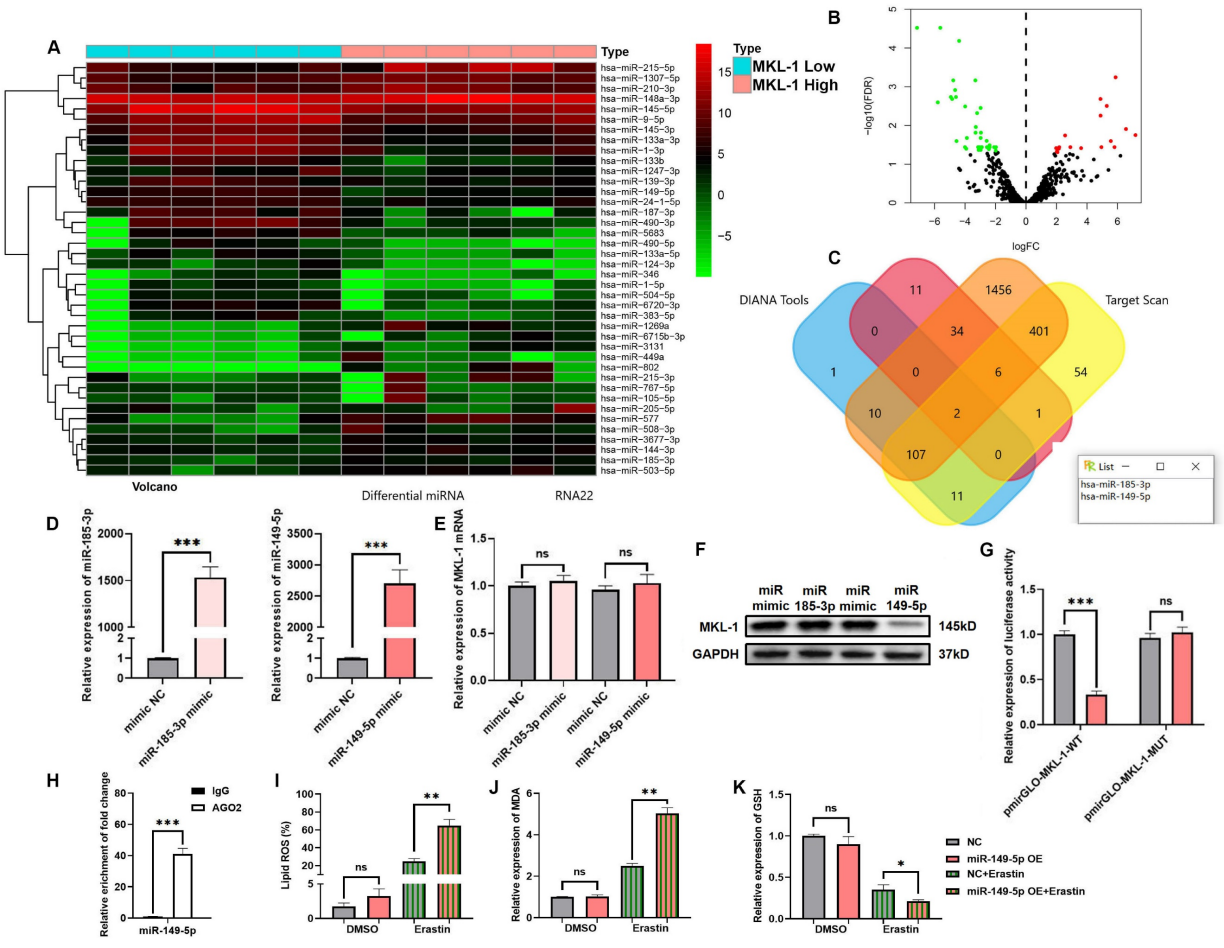
miR-149-5p triggered ferroptosis in gastric cancer via MKL-1 in the presence of ferroptosis inducers. A. The heat map of miRNA micro matrix expression, the miRNA expression was indicated by the colour scale of the heat map as upregulated (red color) and downregulated (green color). Twelve samples were divided into two groups according to whether expression of MKL-1 was high or low. B. The volcano plot of the screened 54 differentially expressed miRNAs. Red represents the 19 miRNAs whose expression was up-regulated. Green represents 35 miRNAs whose expression was down-regulated. C. Venn diagram of miRNAs potentially binding to MKL-1. D. After transfection of mimic NC or miRNA mimic, the miRNA expression in cells were detected by qRT-PCR. E. The mRNA expression of MKL-1 in cells was detected by qRT-PCR. F. The protein expression of MKL-1 in cells was detected by Western Blot. G. After co-transfection of miRNA and luciferase reporter plasmids, luciferase activity in cells was detected by the microplate reader. H. RIP and qRT-PCR were used to verify the binding of AGO2 and miR149-5p. I. After overexpression of miR-149-5p, the Lipid ROS level of cells was detected by C11 BODIPY 581/591 probe and FCM. J. The level of the membrane lipid peroxidation product MDA was detected by the MDA lipid oxidation level detection kit. K. The level of GSH in cells was detected by GSH detection kit. The t-test was used to determine the statistical significance for both groups of analysis. The Fe^2+^ ion, Western blot, Lipid ROS assay, GSH assay NADPH assay, Glu, Gly, L-CYS assay, Luciferase assay and ChIP assay results represented the average values obtained from three independent experiments. The results presented were Mean ± SD. * p < 0.05, ** *p* < 0.01, *** *p* < 0.001, ns = *p* > 0.05.

ncRNAs are RNAs that cannot encode proteins. However, many studies have found that ncRNAs are involved in regulating tumor ferroptosis 23. To screen potential miRNAs that regulate MKL-1. According to the expression of MKL-1, it was divided into two groups: high and low expression. A total of 54 miRNAs that were changed between the two groups were screened, of which 19 were up-regulated and 35 were down-regulated in tumors (Figure 5A-B). Combined with the prediction results of miRNA bioinformatics prediction website Target Scan, DIANA tools and RNA22 (Figure 5C). Two miRNAs that potentially bind to MKL-1 were screened. They were miR-185-3p and miR-149-5p. However, the expression of MKL-1 did not change after transfection of the miR-185-3p mimic in the MGC-803 cell line. Nevertheless, the expression of MKL-1 was significantly reduced after overexpression of miR-149-5p (Figure 5D-F). The dual-luciferase analysis also revealed that only mutating the miR-149-5p binding site on the 3'UTR of MKL-1. The activity of luciferase was no longer reduced (Figure 5G). The above prediction results were further verified by RIP experiments (Figure 5H). Further experiments also showed that in the model constructed by Erastin ferroptosis inducer. Compared with the control group, the levels of Lipid ROS and MDA were up-regulated and the level of GSH was decreased in the miR-149-5p OE group (Figure 5I-J). Moreover, the TCGA database showed that the expression of miR-149-5p was significantly reduced in gastric cancer patients (Figure 5K). The above results showed that restoring the expression of miR-149-5p in cells could significantly enhance the ferroptosis induced by erastin.

### 3.6 Mesenchymal stem cell-derived exosomes reduce the expression of MKL-1 and enhance the anti-tumor effect of ferroptosis inducers

**Figure 6:**
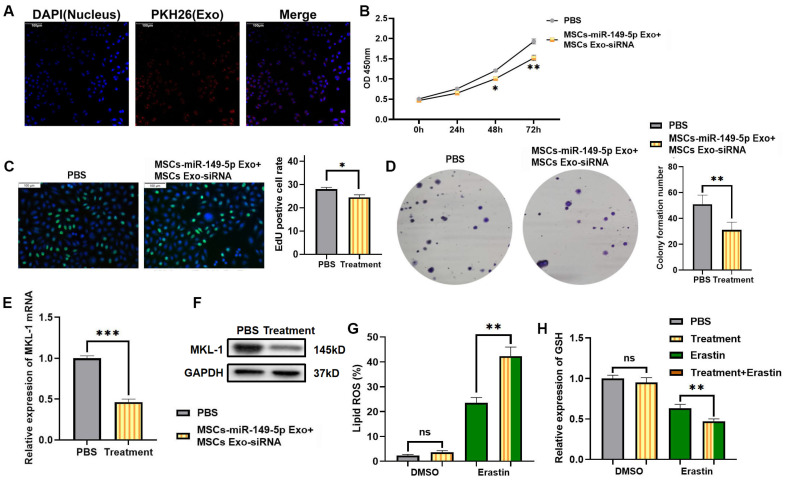
MSCs Exo treatment effect *in vitro*. A. Typical immunofluorescence images of MGC-803 cells after MSCs Exo and MGC-803 cells were co-cultured for 24 hours. DAPI (blue) labelled nuclei and PKH26 (red) labelled exosomes. B. After addition of MSCs-miR-149-5p Exo and MSCs Exo-siRNA treatment, CCK-8 assay was used to detect cell viability after 24, 48 and 72 hours of treatment. C. After 72 hours of MSCs Exo treatment, typical images of EdU-labeled positive cells. D. Clone formation ability of cell line after MSCs Exo treatment. E. After addition of MSCs-miR-149-5p Exo and MSCs Exo-siRNA to cell lines, the mRNA expression of MKL-1 was detected by qRT-PCR. F. After 72 hours of MSCs Exo treatment, the protein expression of MKL-1 in cells was detected by Western Blot. G. The Lipid ROS level of cells was detected by C11 BODIPY 581/591 probe and FCM. H. The level of GSH in cells was detected by GSH detection kit. The t-test was used to determine the statistical significance for both groups of analysis. The CCK-8 assay, EdU assay, clone formation assay, Western blot, qRT-PCR, Lipid ROS assay and GSH assay results represented the average values obtained from three independent experiments. The results presented were Mean ± SD. * *p* < 0.05, ** *p* < 0.01, *** *p* < 0.001, ns = *p* > 0.05.

In view of the above research results, we intend to explore a ferroptosis-based anti-tumor therapy. Furthermore, that was using hUC-MSCs as parent cells. Therapeutic exosomes overexpressing miR-149-5p and encapsulating MKL-1 siRNA were prepared by lentiviral packaging and electroporation combined with the ferroptosis inducer Erastin for combined therapy. First, the characterization of MSCs and exosomes was performed. The results showed no significant difference from the characteristics of MSCs and exosomes reported in the literature 24 (Figure S5, S6). Second, PKH26, a lipophilic dye, was used to stain exosomes. To explore whether MGC-803 cells can take up the extracted exosomes. Red fluorescence appeared in MGC-803 cells as shown by confocal electron microscopy (Figure 6A). This indicates that MGC-803 uptakes exosomes secreted by MSCs. On this basis, exosomes with therapeutic function overexpressing miR-149-5p and encapsulating MKL-1 siRNA were successfully prepared by lentivirus and electroporation (Figure S5).

Next, the therapeutic effects of MSCs Exo were evaluated *in vitro*. The results of the CCK-8 assay, EdU assay and clone formation showed that treatment with MSCs Exo significantly reduced the proliferative capacity of tumor cells (Figure 6B-D) Moreover, it significantly reduced the expression of MKL-1 (Figure 6E-F). Furthermore, in the model constructed with Erastin ferroptosis inducer, the accumulation of Lipid ROS in the treatment group was significantly higher than that in the control group, while less GSH was detected (Figure 6 G-H).

**Figure 7:**
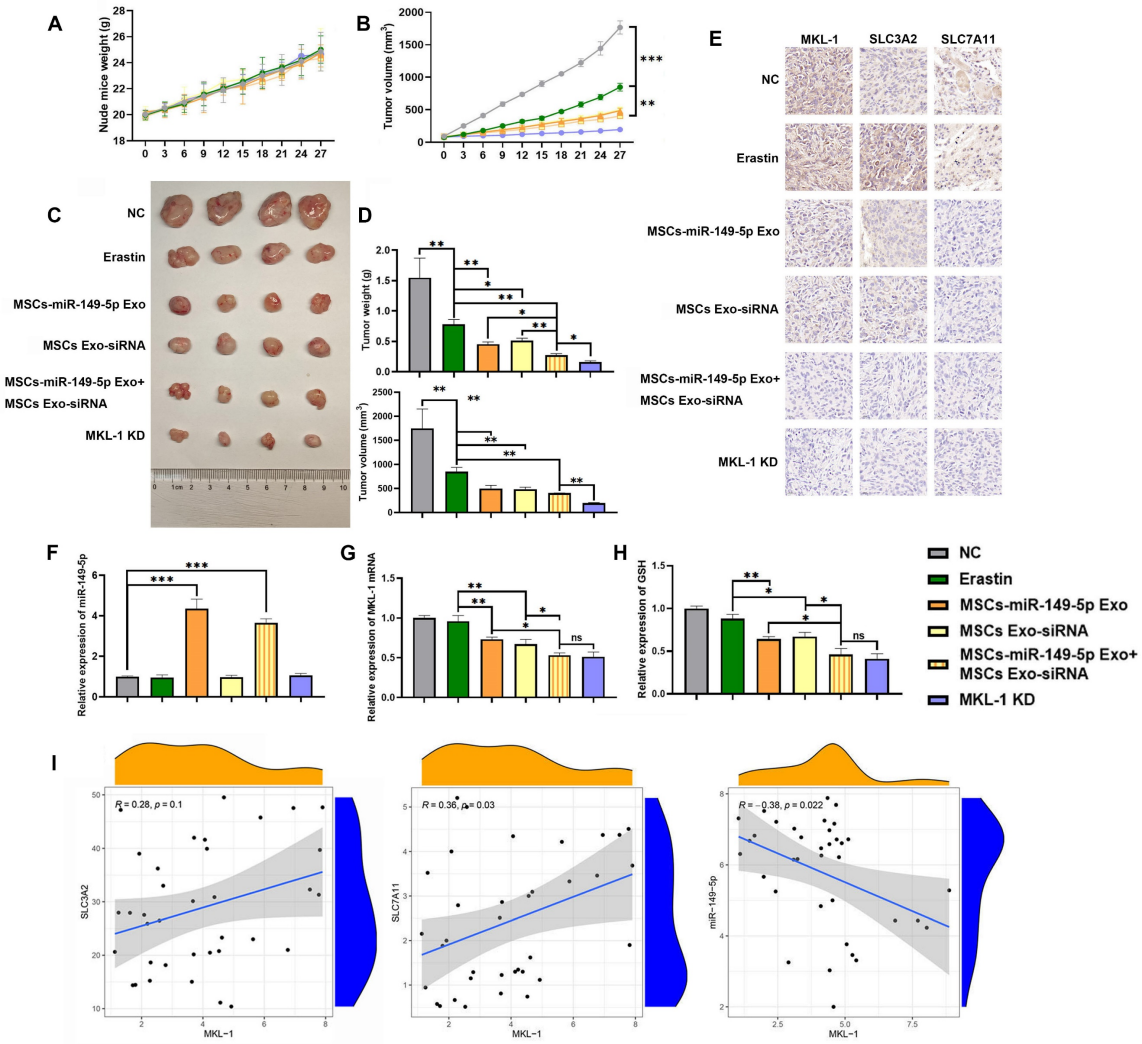
MSCs Exo treatment effect *in vivo*. A. Growth curve of body weight of nude mice after MSCs Exo treatment (n = 4-8 tumors/group). B. Growth curve of subcutaneous tumor volume of nude mice measured *in vitro* after MSCs Exo treatment (n = 4-8 tumors/group). Multiple t-tests (followed by post-hoc test Holm-Sidak) was used to determine the statistical significance against control group. C. Typical images of subcutaneous tumor tissue in nude mice after MSCs Exo treatment. D. Mass and volume of subcutaneous tumor tissue in nude mice after MSCs Exo treatment. E. Typical images of IHC staining of subcutaneous tumor tissue. F. The expression of miR-149-5p in the subcutaneous tumor tissue of nude mice was detected by qRT-PCR. G The mRNA expression of MKL-1 in the subcutaneous tumor tissue of the mouse was detected by qRT-PCR. H. The level of GSH in subcutaneous tumor tissue of nude mice was detected by GSH detection kit. I. Pearson correlation analysis of SLC3A2, SLC7A11, miR-149-5p and MKL-1 mRNA expression in subcutaneous tumor tissue of nude mice. The t-test was used to determine the statistical significance for both groups of analysis (Except for Figure 7I). The results presented were Mean ± SD. * *p* < 0.05, ** *p* < 0.01, *** *p* < 0.001, ns = *p* > 0.05.

The therapeutic potential of MSC-derived exosomes (MSCs Exo) was assessed *in vivo* using a xenograft tumor model in nude mice. Preliminary results indicated a reduction in mean tumor cell count in the group treated with MSC-derived exosomes compared to the PBS group. However, this difference was not statistically significant (Figure S8). Moreover, exosome treatment did not enhance survival in the nude mouse xenograft tumor model until tumors reached the ethical cutoff (Figure S8). Subsequent results from the main experiments demonstrated that the treatment group exhibited a significant restoration of miR-149-5p expression within the tumor, coupled with a decrease in MKL-1 expression. This change was accompanied by an enhanced anti-tumor response to Erastin, as evidenced by reduced tumor growth (Figure 7A-H). Notably, the expression of SLC3A2 and SLC7A11 were also significantly diminished in the treatment group (Figure 7I). The expression of MKL-1 was found to correlate negatively with miR-149-5p and positively with SLC7A11 (Figure 7I). Collectively, our findings suggest that downregulation of MKL-1 notably augments the anti-tumor efficacy of the ferroptosis inducer Erastin in gastric cancer cells, thereby implying a role for MKL-1 in the regulation of ferroptosis in gastric cancer.

## 4. Discussion

Although there has been success in the early clinical diagnosis and treatment of gastric cancer, the overall survival of gastric cancer patients has not been dramatically improved. However, due to drug resistance, the treatment effect of some patients was not very satisfactory. As a new way of programmed cell death, ferroptosis was expected to be a new method of treatment. GPX4 was lower in gastric cancer compared with other cancers, so gastric cancer cells were more prone to ferroptosis than other cells 25. In gastric cancer, some genes are directly involved in regulating ferroptosis. For example, MYB acted as a transcription factor that regulated the expression of GPX4 26. SCD1 promoted gastric cancer tumor growth, migration and ferroptosis resistance 27. The expression of ELOVL5 and FADS1 is up-regulated in mesenchymal gastric cancer cells, and participated in the regulation of ferroptosis by affecting the biosynthesis of PUFA 28. Recent studies have shown that MKL-1 was involved in the regulation of cellular oxidative stress 14, but it was unclear whether MKL-1 was involved in the regulation of cellular ferroptosis. Here, we demonstrate for the first time that MKL-1 is a protein that negatively regulates ferroptosis in gastric cancer cells. Specifically, in the Erastin-induced ferroptosis stress model, the knockdown of MKL-1 significantly increased the production of Lipid ROS and MDA in cells. Furthermore, this phenomenon was not significantly cell-specific. Further research on the molecular mechanism of MKL-1 negatively regulating ferroptosis in gastric cancer cells found that MKL-1 does not directly regulate ferroptosis by affecting the iron metabolism pathway and the production pathway of lipid peroxides. Instead, it promoted its transcription by binding to the CArG Box-specific binding site in the promoters of SLC3A2 and SLC7A11, increasing the level of GSH in cells, thereby preventing cells from ferroptosis by enhancing the ability of cells to decompose lipid peroxides. It is worth mentioning that MKL-1 is widely expressed in a variety of tissues 29. And often its overexpression was closely related to the poor prognosis of patients 30. However, MKL-1 knockout mice resulted in embryonic death in some mice 18. Therefore, the MKL-1 knockout model was not used in this study. In addition, research by others in our group found that overexpression of MKL-1 increased the transcriptional activity of the transcription factor NRF2 through the formation of the MKL-1/SRF complex 31. Numerous studies have shown that NRF2, a transcription factor, was considered to be a key regulator of cellular antioxidant responses. Because many of its downstream target genes were involved in preventing or correcting redox imbalance in cells 32. In the present study, we only found that MKL-1 regulates ferroptosis by affecting the system Xc-, exogenous cystine supplementation completely blocked the effect of knockdown of MKL-1. However, the mechanism by which MKL-1 affects ferroptosis through NRF2 has not been elucidated. According to our experience, a life activity is affected by multiple regulatory mechanisms. Therefore, elucidating the underlying mechanism will be the focus of future research.

In recent years, miR-149 has received extensive attention from researchers around the world. miR-149 has been shown to play different roles in different cancers. For example, Feng et al. 33 showed that miR-149 inhibited liver cancer progression by targeting TRADD. In lung cancer, miR-149 reduces the proliferation and invasive ability of lung cancer cells by directly targeting the 3'UTR region of B3GNT3 34. In ovarian cancer, miR-149 inhibits ovarian cancer cell proliferation and migration by regulating the expression of MSI2 35. To date, reports on miR-149 have shown that its expression is reduced in most cancers and functions as a tumor suppressor. Hence, the expression profiles of miRNAs of STAD in the TCGA database were downloaded, and MiR-149-5p was found to be down-regulated in gastric cancer. This is basically consistent with the report by Li et al. 36 that miR-149 is down-regulated in gastric cancer cells. However, there is no report that miR-149-5p is involved in the regulation of ferroptosis. Therefore, gastric cancer cell line MGC-803 with stable overexpression of miR-149-5p was constructed by lentiviral packaging technology. It was found that overexpression of miR-149-5p significantly reduced the expression of MKL-1 and enhanced the occurrence of ferroptosis induced by Erastin. It provided key experimental evidence for promoting the anti-tumor effect of ferroptosis inducers by interfering with the expression of MKL-1.

Mesenchymal stem cells were a type of pluripotent stem cells. They were isolated from fetal or adult tissues, including adipose tissue, bone marrow, and umbilical cord blood 37. MSCs have been widely used in the treatment of various human diseases 38. Such as limb ischemia, skin wounds and cartilage defects 39. However, there were problems in carcinogenicity and tissue rejection due to the urgent treatment of MSCs, which greatly limited the clinical application of MSCs 40. Therefore, we focused our attention on the exosomes secreted by MSCs. Exosomes secreted by MSCs acted as a vehicle for cell-to-cell delivery, affecting their proliferation, metastasis, and angiogenesis by transporting intravesicular biomolecules to target cells 41. However, exosomes sometimes played a tumor-promoting role in tumors 42, and sometimes played an anti-tumor role in tumors 43. Notably, exosomes secreted by MSCs derived from different tissues had different effects on tumors. A meta-analysis showed that only 26% and 46% of studies on bone marrow-derived mesenchymal stem cells and adipose-derived mesenchymal stem cells were associated with tumor suppression, while 88% of studies on umbilical cord mesenchymal stem cells showed that they have ability to suppress tumors 44. Therefore, in our study, umbilical cord mesenchymal stem cells were selected as MSCs for producing exosomes with therapeutic functions. Because the MSCs used were not isolated and cultured by our laboratory. Before starting to prepare exosomes with therapeutic function, the expression of MSCs markers CD90 and CD105 was analyzed by FCM, and the differentiation experiment was induced to determine the purity of MSCs. The results showed that the MSCs used in our study comply with the criteria of the International Society for Cell Therapy for identifying MSCs 45.

Next, two methods were used to generate therapeutic exosomes overexpressing miR-149-5p and encapsulating MKL-1 siRNA. O'Brien et al. 46 used a lentiviral system to successfully generate miR-379-enriched exosomes. Mendt et al. 47 also successfully used electroporation to deliver KRAS G12D siRNA into exosomes produced by MSCs. Then, miR-149-5p was overexpressed in MSCs by lentiviral packaging system to generate the desired exosomes. The siRNA of MKL-1 was introduced into exosomes by electroporation. Finally, exosomes with therapeutic function were prepared.

In order to verify the therapeutic effect of the prepared exosomes, we performed validation *in vitro* and *in vivo*, respectively. *In vitro* experiments showed that the proliferative capacity of the cells in the treatment group was significantly inhibited. And significantly enhanced the anti-tumor effect of Erastin. Further, it has been reported in the literature that the delivery of intravesicular substances via exosomes was plagued by misplaced accumulation. The major retention of exosomes occured mainly in the liver and spleen, which contain large numbers of macrophages due to endocytosis 48. Therefore, injection of exosomes into tumors was chosen. The results showed that the expression of miR-149-5p was up-regulated in the exosome-treated group, while the expression of MKL-1 was inhibited, enhancing the anti-tumor effect of Erastin.

However, research on ferroptosis is currently quite limited. There are still many unanswered questions. It has been found through literature reports that these cell deaths share some common upstream mechanisms, such as p53. Furthermore, the redox function of cellular iron in ferroptosis has not been completely ruled out. At the same time, this study only explored the role of MKL-1 in regulating ferroptosis in the gastric cancer ferroptosis stress model established by the ferroptosis inducer Erastin. Whether this regulatory phenomenon is cancer-specific or is affected by the level of ferroptosis remains to be further explored. Meanwhile, like ferroptosis, the expression of MKL-1 is also regulated in multiple ways. It is now known that a gene may be regulated by multiple miRNAs, and our study only found an effect of miR-149-5p on the expression of MKL-1. It is certain that there are other miRNAs, lncRNAs or other epigenetic modifications that affect the expression of MKL-1. Otherwise, the anti-tumor effect of exosomes with therapeutic function was isolated and prepared by using the exosome isolation kit, and its anti-tumor effect was investigated by intratumoral injection. This method of evaluating its therapeutic effect is still very single. In the real process of tumor treatment, multiple methods are often used in combination therapy. However, whether the use of exosome therapy will produce antagonism with other treatments and the appropriate way of administration, dosage are still unclear. These discussions still require extensive experiments to further explore. We have reason to believe that, with the deepening of research and the advancement of science and technology, the above problems will be solved in the near future.

**Figure 8:**
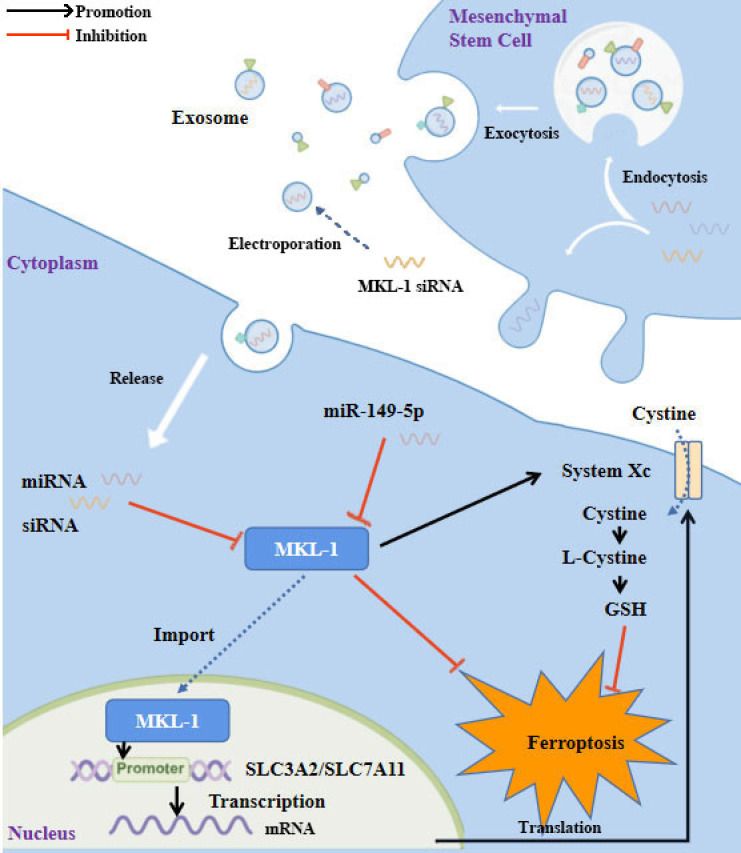
Study flowchart. MKL-1, functioning as a transcription factor, promoted the transcription of SLC3A2 and SLC7A11, the pivotal proteins of System Xc-. This led to an increase in the intracellular levels of glutathione (GSH), which in turn protected cancer cells from ferroptotic stress. Expression of MKL-1 was found to be suppressed by miR-149-5p, which also contributed to the process of ferroptosis in gastric cancer cells via this pathway. A further enhancement in the therapeutic effect of ferroptosis inducers on gastric cancer cells was achieved by constructing exosomes that overexpressed miR-149-5p and simultaneously transfected MKL-1 siRNA.

In conclusion, as shown in the figure (Figure 8), this study identified MKL-1 as a negative regulator of ferroptosis. MKL-1 affected the synthesis of GSH through System Xc-, and ultimately reduced the level of lipid peroxidation in cells and reduced the occurrence of cellular ferroptosis. A novel anti-tumor idea combining MSCs-secreted exosomes with ferroptosis inducers was developed. These observations provided new insights into the clinical treatment of gastric cancer.

## Supplementary Material

Supplementary figures.

## References

[1] Sung H, Ferlay J, Siegel RL, Laversanne M, Soerjomataram I, Jemal A, Bray F (2021). Global Cancer Statistics 2020: GLOBOCAN Estimates of Incidence and Mortality Worldwide for 36 Cancers in 185 Countries. *CA Cancer J Clin*.

[2] Rawla P, Barsouk A (2019). Epidemiology of gastric cancer: global trends, risk factors and prevention. *Prz Gastroenterol*.

[3] Macdonald JS, Smalley SR, Benedetti J, Hundahl SA, Estes NC, Stemmermann GN, Haller DG, Ajani JA, Gunderson LL, Jessup JM (2001). Chemoradiotherapy after surgery compared with surgery alone for adenocarcinoma of the stomach or gastroesophageal junction. *N Engl J Med*.

[4] Fuchs CS, Doi T, Jang RW, Muro K, Satoh T, Machado M, Sun W, Jalal SI, Shah MA, Metges JP (2018). Safety and Efficacy of Pembrolizumab Monotherapy in Patients With Previously Treated Advanced Gastric and Gastroesophageal Junction Cancer: Phase 2 Clinical KEYNOTE-059 Trial. *JAMA Oncol*.

[5] Bang YJ, Van Cutsem E, Feyereislova A, Chung HC, Shen L, Sawaki A, Lordick F, Ohtsu A, Omuro Y, Satoh T (2010). Trastuzumab in combination with chemotherapy versus chemotherapy alone for treatment of HER2-positive advanced gastric or gastro-oesophageal junction cancer (ToGA): a phase 3, open-label, randomised controlled trial. *Lancet*.

[6] Wilke H, Muro K, Van Cutsem E, Oh SC, Bodoky G, Shimada Y, Hironaka S, Sugimoto N, Lipatov O, Kim TY (2014). Ramucirumab plus paclitaxel versus placebo plus paclitaxel in patients with previously treated advanced gastric or gastro-oesophageal junction adenocarcinoma (RAINBOW): a double-blind, randomised phase 3 trial. *Lancet Oncol*.

[7] Chen Z, Li Y, Tan B, Zhao Q, Fan L, Li F, Zhao X (2020). Progress and current status of molecule-targeted therapy and drug resistance in gastric cancer. *Drugs Today (Barc)*.

[8] Spirina LV, Avgustinovich AV, Afanas'ev SG, Cheremisina OV, Volkov MY, Choynzonov EL, Gorbunov AK, Usynin EA (2020). Molecular Mechanism of Resistance to Chemotherapy in Gastric Cancers, the Role of Autophagy. *Curr Drug Targets*.

[9] Al Dhaheri Y, Attoub S, Ramadan G, Arafat K, Bajbouj K, Karuvantevida N, AbuQamar S, Eid A, Iratni R (2014). Carnosol induces ROS-mediated beclin1-independent autophagy and apoptosis in triple negative breast cancer. *PLoS One*.

[10] Dixon SJ, Lemberg KM, Lamprecht MR, Skouta R, Zaitsev EM, Gleason CE, Patel DN, Bauer AJ, Cantley AM, Yang WS (2012). Ferroptosis: an iron-dependent form of nonapoptotic cell death. *Cell*.

[11] Martin-Sanchez D, Ruiz-Andres O, Poveda J, Carrasco S, Cannata-Ortiz P, Sanchez-Nino MD, Ruiz Ortega M, Egido J, Linkermann A, Ortiz A (2017). Ferroptosis, but Not Necroptosis, Is Important in Nephrotoxic Folic Acid-Induced AKI. *J Am Soc Nephrol*.

[12] Lu L, Chen B, Xu Y, Zhang X, Jin L, Qian H, Wang Y, Liang ZF (2022). Role of ferroptosis and ferroptosis-related non-coding RNAs in the occurrence and development of gastric cancer. *Front Pharmacol*.

[13] Di Marco E, Aiello F, Lombardo M, Di Marino M, Missiroli F, Mancino R, Ricci F, Nucci C, Noce A, Di Daniele N (2022). A literature review of hypertensive retinopathy: systemic correlations and new technologies. *Eur Rev Med Pharmacol Sci*.

[14] Alvarez SW, Sviderskiy VO, Terzi EM, Papagiannakopoulos T, Moreira AL, Adams S, Sabatini DM, Birsoy K, Possemato R (2017). NFS1 undergoes positive selection in lung tumours and protects cells from ferroptosis. *Nature*.

[15] Zhang C, Liu X, Jin S, Chen Y, Guo R (2022). Ferroptosis in cancer therapy: a novel approach to reversing drug resistance. *Mol Cancer*.

[16] Ma Z, Morris SW, Valentine V, Li M, Herbrick JA, Cui X, Bouman D, Li Y, Mehta PK, Nizetic D (2001). Fusion of two novel genes, RBM15 and MKL1, in the t(1;22)(p13;q13) of acute megakaryoblastic leukemia. *Nat Genet*.

[17] Mercher T, Coniat MB, Monni R, Mauchauffe M, Nguyen Khac F, Gressin L, Mugneret F, Leblanc T, Dastugue N, Berger R (2001). Involvement of a human gene related to the Drosophila spen gene in the recurrent t(1;22) translocation of acute megakaryocytic leukemia. *Proc Natl Acad Sci U S A*.

[18] Manabe I, Owens GK (2001). CArG elements control smooth muscle subtype-specific expression of smooth muscle myosin *in vivo*. *J Clin Invest*.

[19] Miano JM (2003). Serum response factor: toggling between disparate programs of gene expression. *J Mol Cell Cardiol*.

[20] McDonald OG, Wamhoff BR, Hoofnagle MH, Owens GK (2006). Control of SRF binding to CArG box chromatin regulates smooth muscle gene expression *in vivo*. *J Clin Invest*.

[21] Sprenkeler EGG, Guenther C, Faisal I, Kuijpers TW, Fagerholm SC (2021). Molecular Mechanisms of Leukocyte Migration and Its Potential Targeting-Lessons Learned From MKL1/SRF-Related Primary Immunodeficiency Diseases. *Front Immunol*.

[22] Li H, Li HH, Chen Q, Wang YY, Fan CC, Duan YY, Huang Y, Zhang HM, Li JP, Zhang XY (2022). miR-142-5p Inhibits Cell Invasion and Migration by Targeting DNMT1 in Breast Cancer. *Oncol Res*.

[23] Liu Z, Sun J, Li C, Xu L, Liu J (2021). MKL1 regulates hepatocellular carcinoma cell proliferation, migration and apoptosis via the COMPASS complex and NF-kappaB signaling. *BMC Cancer*.

[24] Zhang HM, Li H, Wang GX, Wang J, Xiang Y, Huang Y, Shen C, Dai ZT, Li JP, Zhang TC (2020). MKL1/miR-5100/CAAP1 loop regulates autophagy and apoptosis in gastric cancer cells. *Neoplasia*.

[25] Sobierajska K, Wawro ME, Niewiarowska J (2022). Oxidative Stress Enhances the TGF-beta2-RhoA-MRTF-A/B Axis in Cells Entering Endothelial-Mesenchymal Transition. *Int J Mol Sci*.

[26] Stockwell BR (2022). Ferroptosis turns 10: Emerging mechanisms, physiological functions, and therapeutic applications. *Cell*.

[27] Wahlgren J, De LKT, Brisslert M, Vaziri Sani F, Telemo E, Sunnerhagen P, Valadi H (2012). Plasma exosomes can deliver exogenous short interfering RNA to monocytes and lymphocytes. *Nucleic Acids Res*.

[28] Xiang Y, Liao XH, Yu CX, Yao A, Qin H, Li JP, Hu P, Li H, Guo W, Gu CJ (2017). MiR-93-5p inhibits the EMT of breast cancer cells via targeting MKL-1 and STAT3. *Exp Cell Res*.

[29] Dai ZT, Xiang Y, Duan YY, Wang J, Li JP, Zhang HM, Cheng C, Wang Q, Zhang TC, Liao XH (2021). MiR-17-5p and MKL-1 modulate stem cell characteristics of gastric cancer cells. *Int J Biol Sci*.

[30] Sun Y, Boyd K, Xu W, Ma J, Jackson CW, Fu A, Shillingford JM, Robinson GW, Hennighausen L, Hitzler JK (2006). Acute myeloid leukemia-associated Mkl1 (Mrtf-a) is a key regulator of mammary gland function. *Mol Cell Biol*.

[31] Doll S, Proneth B, Tyurina YY, Panzilius E, Kobayashi S, Ingold I, Irmler M, Beckers J, Aichler M, Walch A (2017). ACSL4 dictates ferroptosis sensitivity by shaping cellular lipid composition. *Nat Chem Biol*.

[32] Muckenthaler MU, Rivella S, Hentze MW, Galy B (2017). A Red Carpet for Iron Metabolism. *Cell*.

[33] Seibt TM, Proneth B, Conrad M (2019). Role of GPX4 in ferroptosis and its pharmacological implication. *Free Radic Biol Med*.

[34] Bersuker K, Hendricks JM, Li Z, Magtanong L, Ford B, Tang PH, Roberts MA, Tong B, Maimone TJ, Zoncu R (2019). The CoQ oxidoreductase FSP1 acts parallel to GPX4 to inhibit ferroptosis. *Nature*.

[35] Ursini F, Maiorino M (2020). Lipid peroxidation and ferroptosis: The role of GSH and GPx4. *Free Radic Biol Med*.

[36] Capelletti MM, Manceau H, Puy H, Peoc'h K (2020). Ferroptosis in Liver Diseases: An Overview. *Int J Mol Sci*.

[37] Koppula P, Zhuang L, Gan B (2021). Cystine transporter SLC7A11/xCT in cancer: ferroptosis, nutrient dependency, and cancer therapy. *Protein Cell*.

[38] Zhang H, Deng T, Liu R, Ning T, Yang H, Liu D, Zhang Q, Lin D, Ge S, Bai M (2020). CAF secreted miR-522 suppresses ferroptosis and promotes acquired chemo-resistance in gastric cancer. *Mol Cancer*.

[39] Ni H, Qin H, Sun C, Liu Y, Ruan G, Guo Q, Xi T, Xing Y, Zheng L (2021). MiR-375 reduces the stemness of gastric cancer cells through triggering ferroptosis. *Stem Cell Res Ther*.

[40] Luo M, Wu L, Zhang K, Wang H, Zhang T, Gutierrez L, O'Connell D, Zhang P, Li Y, Gao T (2018). miR-137 regulates ferroptosis by targeting glutamine transporter SLC1A5 in melanoma. *Cell Death Differ*.

[41] Zhang P, Zhou X, He M, Shang Y, Tetlow AL, Godwin AK, Zeng Y (2019). Ultrasensitive detection of circulating exosomes with a 3D-nanopatterned microfluidic chip. *Nat Biomed Eng*.

[42] Yong T, Zhang X, Bie N, Zhang H, Zhang X, Li F, Hakeem A, Hu J, Gan L, Santos HA (2019). Tumor exosome-based nanoparticles are efficient drug carriers for chemotherapy. *Nat Commun*.

[43] Lu M, Xing H, Xun Z, Yang T, Ding P, Cai C, Wang D, Zhao X (2018). Exosome-based small RNA delivery: Progress and prospects. *Asian J Pharm Sci*.

[44] Zhao L, Peng Y, He S, Li R, Wang Z, Huang J, Lei X, Li G, Ma Q (2021). Apatinib induced ferroptosis by lipid peroxidation in gastric cancer. *Gastric Cancer*.

[45] Hao S, Yu J, He W, Huang Q, Zhao Y, Liang B, Zhang S, Wen Z, Dong S, Rao J (2017). Cysteine Dioxygenase 1 Mediates Erastin-Induced Ferroptosis in Human Gastric Cancer Cells. *Neoplasia*.

[46] Wang C, Shi M, Ji J, Cai Q, Zhao Q, Jiang J, Liu J, Zhang H, Zhu Z, Zhang J (2020). Stearoyl-CoA desaturase 1 (SCD1) facilitates the growth and anti-ferroptosis of gastric cancer cells and predicts poor prognosis of gastric cancer. *Aging (Albany NY)*.

[47] Lee JY, Nam M, Son HY, Hyun K, Jang SY, Kim JW, Kim MW, Jung Y, Jang E, Yoon SJ (2020). Polyunsaturated fatty acid biosynthesis pathway determines ferroptosis sensitivity in gastric cancer. *Proc Natl Acad Sci U S A*.

[48] Yu Y, Xie Y, Cao L, Yang L, Yang M, Lotze MT, Zeh HJ, Kang R, Tang D (2015). The ferroptosis inducer erastin enhances sensitivity of acute myeloid leukemia cells to chemotherapeutic agents. *Mol Cell Oncol*.

[49] Xu Y, Luo Y, Wang ZY, Li X, Zheng P, Zhang TC (2017). MRTF-A can activate Nrf2 to increase the resistance to doxorubicin. *Oncotarget*.

[50] Dodson M, Castro-Portuguez R, Zhang DD (2019). NRF2 plays a critical role in mitigating lipid peroxidation and ferroptosis. *Redox Biol*.

[51] Feng Q, Zhang H, Nie X, Li Y, Chen WD, Wang YD (2020). miR-149* Suppresses Liver Cancer Progression by Down-Regulating Tumor Necrosis Factor Receptor 1-Associated Death Domain Protein Expression. *Am J Pathol*.

[52] Sun Y, Liu T, Xian L, Liu W, Liu J, Zhou H (2020). B3GNT3, a Direct Target of miR-149-5p, Promotes Lung Cancer Development and Indicates Poor Prognosis of Lung Cancer. *Cancer Manag Res*.

[53] Zhao LW, Yu AJ, Zhang YJ, Wang XC, Han B, Wang XH (2020). MicroRNA-149 suppresses the malignant phenotypes of ovarian cancer via downregulation of MSI2 and inhibition of PI3K/AKT pathway. *Eur Rev Med Pharmacol Sci*.

[54] Li P, Shan JX, Chen XH, Zhang D, Su LP, Huang XY, Yu BQ, Zhi QM, Li CL, Wang YQ (2015). Epigenetic silencing of microRNA-149 in cancer-associated fibroblasts mediates prostaglandin E2/interleukin-6 signaling in the tumor microenvironment. *Cell Res*.

[55] Squillaro T, Peluso G, Galderisi U (2016). Clinical Trials With Mesenchymal Stem Cells: An Update. *Cell Transplant*.

[56] Minguell JJ, Erices A, Conget P (2001). Mesenchymal stem cells. *Exp Biol Med (Maywood)*.

[57] Ryu DJ, Jeon YS, Park JS, Bae GC, Kim JS, Kim MK (2020). Comparison of Bone Marrow Aspirate Concentrate and Allogenic Human Umbilical Cord Blood Derived Mesenchymal Stem Cell Implantation on Chondral Defect of Knee: Assessment of Clinical and Magnetic Resonance Imaging Outcomes at 2-Year Follow-Up. *Cell Transplant*.

[58] Nammian P, Asadi-Yousefabad SL, Daneshi S, Sheikhha MH, Tabei SMB, Razban V (2021). Comparative analysis of mouse bone marrow and adipose tissue mesenchymal stem cells for critical limb ischemia cell therapy. *Stem Cell Res Ther*.

[59] Maharlooei MK, Bagheri M, Solhjou Z, Jahromi BM, Akrami M, Rohani L, Monabati A, Noorafshan A, Omrani GR (2011). Adipose tissue derived mesenchymal stem cell (AD-MSC) promotes skin wound healing in diabetic rats. *Diabetes Res Clin Pract*.

[60] Xia C, Dai Z, Jin Y, Chen P (2021). Emerging Antioxidant Paradigm of Mesenchymal Stem Cell-Derived Exosome Therapy. *Front Endocrinol (Lausanne)*.

[61] Veceric-Haler Z, Cerar A, Perse M (2017). (Mesenchymal) Stem Cell-Based Therapy in Cisplatin-Induced Acute Kidney Injury Animal Model: Risk of Immunogenicity and Tumorigenicity. *Stem Cells Int*.

[62] Barkholt L, Flory E, Jekerle V, Lucas-Samuel S, Ahnert P, Bisset L, Buscher D, Fibbe W, Foussat A, Kwa M (2013). Risk of tumorigenicity in mesenchymal stromal cell-based therapies-bridging scientific observations and regulatory viewpoints. *Cytotherapy*.

[63] Roccaro AM, Sacco A, Maiso P, Azab AK, Tai YT, Reagan M, Azab F, Flores LM, Campigotto F, Weller E (2013). BM mesenchymal stromal cell-derived exosomes facilitate multiple myeloma progression. *J Clin Invest*.

[64] Zhang F, Guo J, Zhang Z, Qian Y, Wang G, Duan M, Zhao H, Yang Z, Jiang X (2022). Mesenchymal stem cell-derived exosome: A tumor regulator and carrier for targeted tumor therapy. *Cancer Lett*.

[65] Grange C, Tapparo M, Collino F, Vitillo L, Damasco C, Deregibus MC, Tetta C, Bussolati B, Camussi G (2011). Microvesicles released from human renal cancer stem cells stimulate angiogenesis and formation of lung premetastatic niche. *Cancer Res*.

[66] Fonsato V, Collino F, Herrera MB, Cavallari C, Deregibus MC, Cisterna B, Bruno S, Romagnoli R, Salizzoni M, Tetta C (2012). Human liver stem cell-derived microvesicles inhibit hepatoma growth in SCID mice by delivering antitumor microRNAs. *Stem Cells*.

[67] Christodoulou I, Goulielmaki M, Devetzi M, Panagiotidis M, Koliakos G, Zoumpourlis V (2018). Mesenchymal stem cells in preclinical cancer cytotherapy: a systematic review. *Stem Cell Res Ther*.

[68] Dominici M, Le Blanc K, Mueller I, Slaper-Cortenbach I, Marini F, Krause D, Deans R, Keating A, Prockop D, Horwitz E (2006). Minimal criteria for defining multipotent mesenchymal stromal cells. The International Society for Cellular Therapy position statement. *Cytotherapy*.

[69] O'Brien KP, Khan S, Gilligan KE, Zafar H, Lalor P, Glynn C, O'Flatharta C, Ingoldsby H, Dockery P, De Bhulbh A (2018). Employing mesenchymal stem cells to support tumor-targeted delivery of extracellular vesicle (EV)-encapsulated microRNA-379. *Oncogene*.

[70] Mendt M, Kamerkar S, Sugimoto H, McAndrews KM, Wu CC, Gagea M, Yang S, Blanko EVR, Peng Q, Ma X (2018). Generation and testing of clinical-grade exosomes for pancreatic cancer. *JCI Insight*.

[71] Chen H, Xue R, Huang P, Wu Y, Fan W, He X, Dong Y, Liu C (2022). Modified Exosomes: a Good Transporter for miRNAs within Stem Cells to Treat Ischemic Heart Disease. *J Cardiovasc Transl Res*.

