# Supplementary material for: MKL-1 suppresses ferroptosis by activating system Xc- and increasing glutathione synthesis
Source: Int J Biol Sci. 2023 Aug 21;19(14):4457–75. doi: 10.7150/ijbs.80666 (PMC10535709; doi:10.7150/ijbs.80666)
Supplement: Supplementary file 1 — Supplementary figures. [file ijbsv19p4457s1.pdf]

MKL-1 suppresses ferroptosis by activating system Xc- and increasing glutathione synthesis

Supplementary materials

Zhou-Tong Dai[1,2,3], Yong-Lin Wu[4], Xing-Rui Li[4*], Xing-Hua Liao[3*]

1 Department of Gynaecological Oncology, Tongji Hospital, Tongji Medical College, Huazhong University of Science and Technology, Wuhan, China.

2 National Clinical Research Centre for Obstetrics and Gynaecology, Cancer Biology Research Centre (Key Laboratory of the Ministry of Education), Tongji Hospital, Tongji Medical College, Huazhong University of Science and Technology, Wuhan, China.

3 Institute of Biology and Medicine, College of Life and Health Science, Wuhan University of Science and Technology, Wuhan, China.

4 Department of Thyroid and Breast Surgery, Tongji Hospital, Tongji Medical College, Huazhong University of Science and Technology, Wuhan, China

[*]Corresponding author: Xing-Rui Li, Department of Thyroid and Breast Surgery, Tongji Hospital, Tongji Medical College, Huazhong University of Science and Technology, 430030, Wuhan, China, E-mail: lixingrui@tjh.tjmu.edu.cn; Xing-Hua Liao, Institute of Biology and Medicine, College of Life and Health Science, Wuhan University of Science and Technology, Wuhan, 430070, China, E-mail: xinghualiao@hotmail.com.

Figure S1

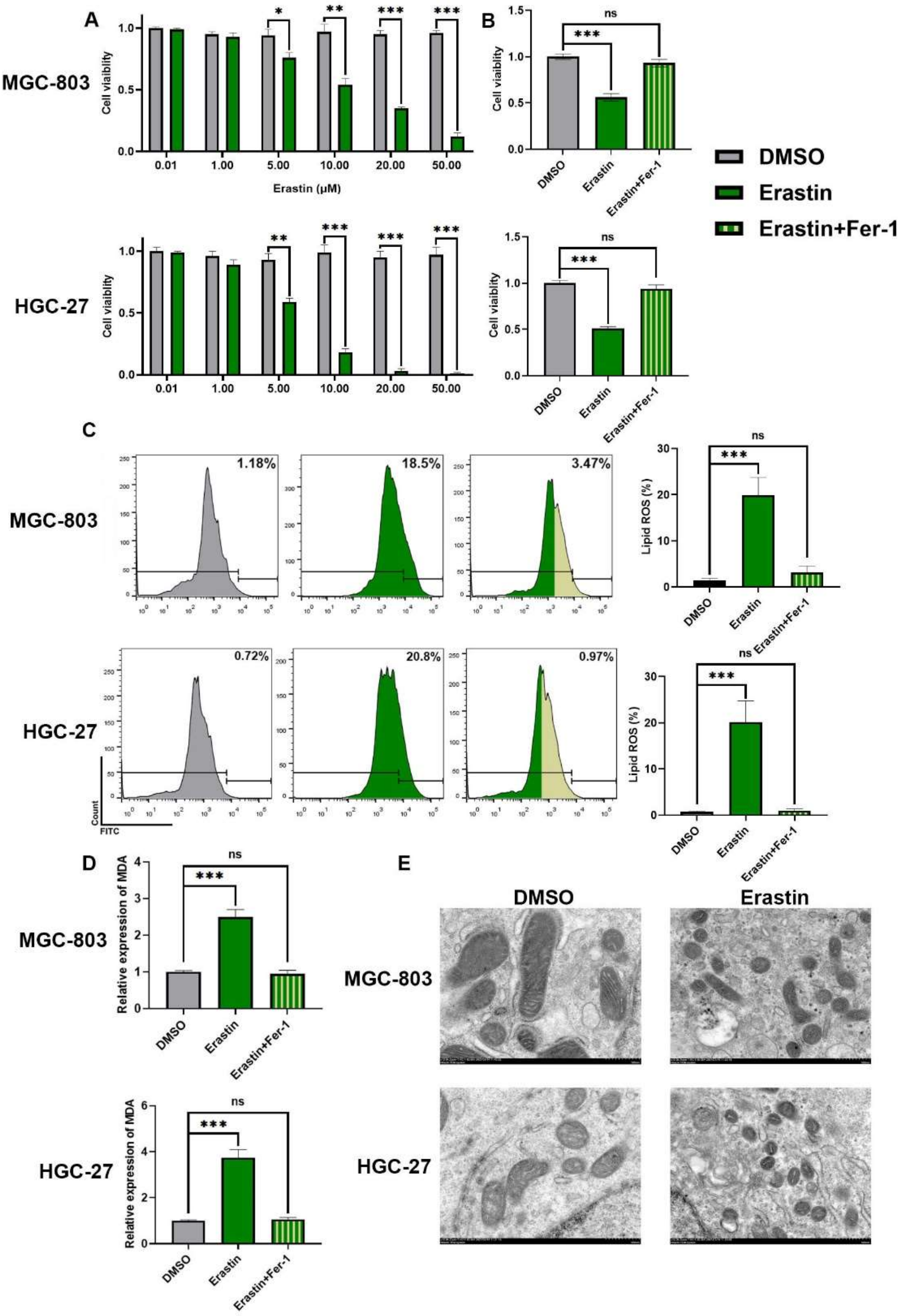
A
B
C
D
E
MGC-803
HGC-27
Cell viability
Erastin (μM)
0.01
1.00
5.00
10.00
20.00
50.00
DMSO
Erastin
Erastin+Fer-1
ns
1.18%
18.5%
3.47%
0.72%
20.8%
0.97%
Count
FITC
Lipid ROS (%)
Relative expression of MDA

Figure S2

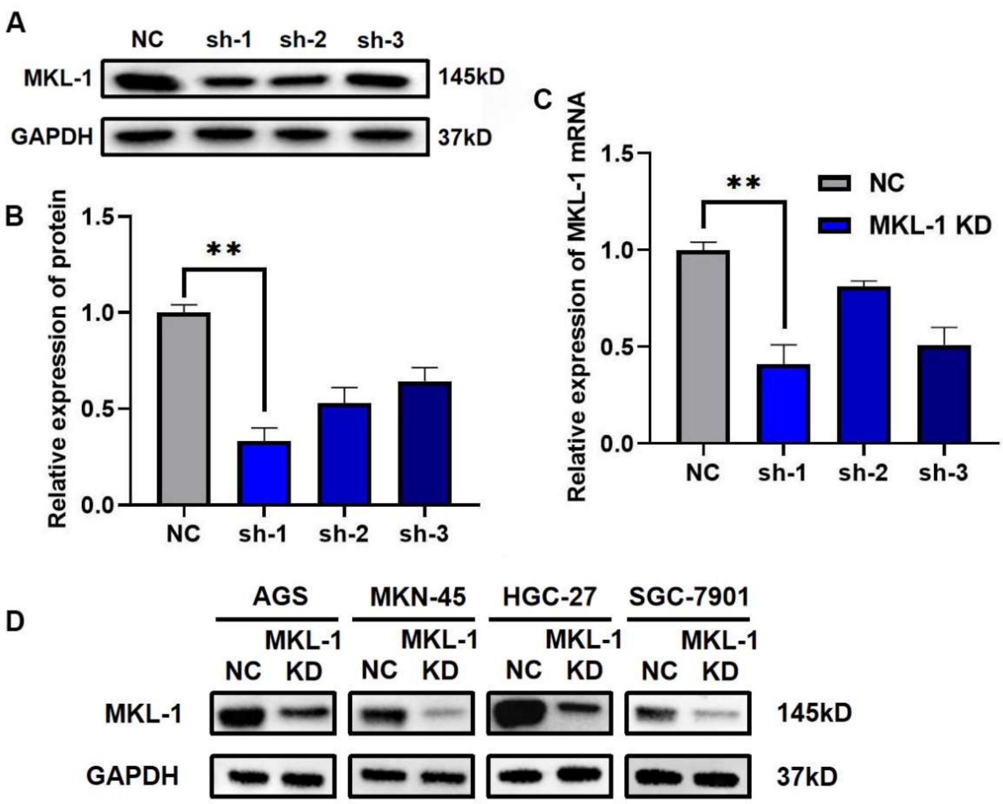
A
NC
sh-1
sh-2
sh-3
MKL-1
145kD
GAPDH
37kD
B
Relative expression of protein
1.5
1.0
0.5
0.0
**
NC
sh-1
sh-2
sh-3
C
Relative expression of MKL-1 mRNA
1.5
1.0
0.5
0.0
**
NC
MKL-1 KD
NC
sh-1
sh-2
sh-3
D
AGS
MKN-45
HGC-27
SGC-7901
MKL-1
NC
KD
MKL-1
145kD
GAPDH
37kD

Figure S3

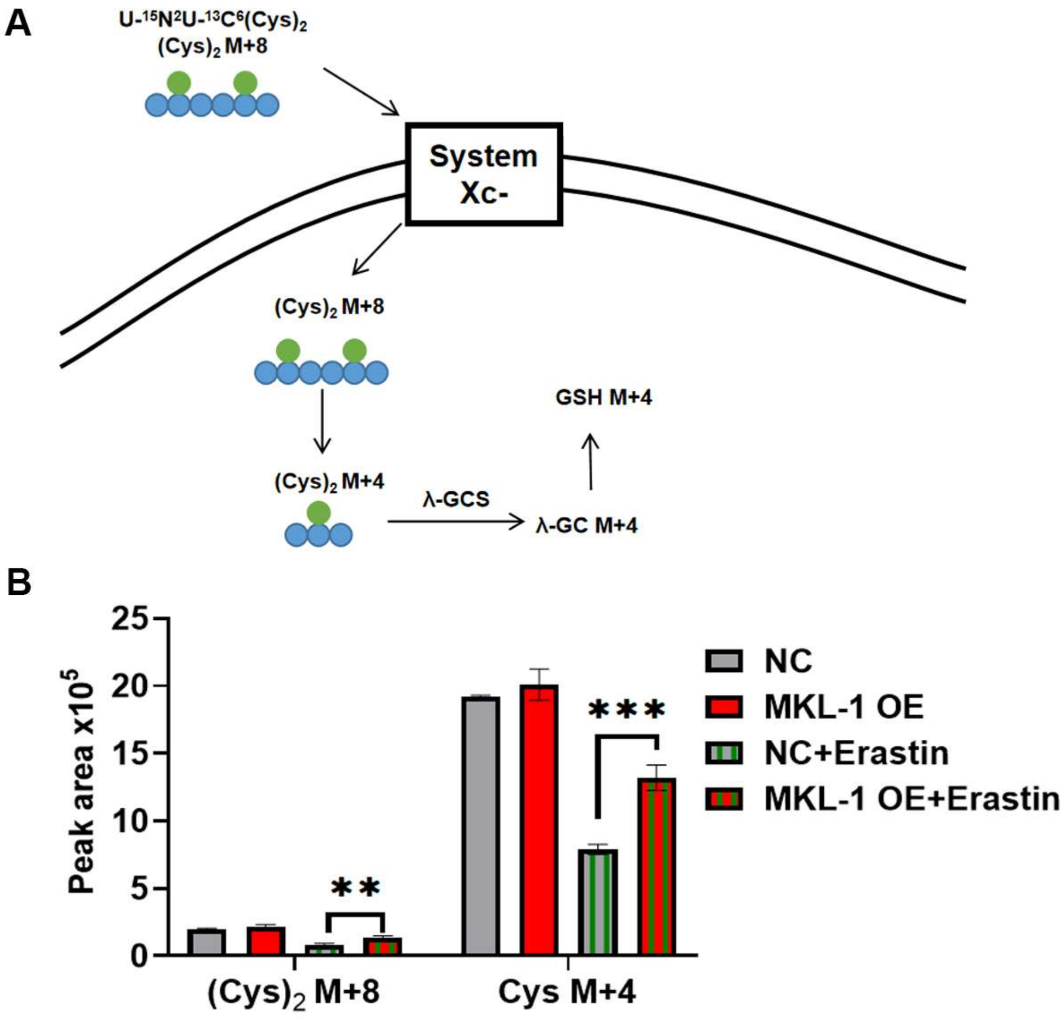
A
U-15N2U-13C6(Cys)2
(Cys)2 M+8
System Xc-
(Cys)2 M+8
GSH M+4
(Cys)2 M+4
λ-GCS
λ-GC M+4
B
Peak area x10^5
25
20
15
10
5
0
**
***
(Cys)2 M+8
Cys M+4
NC
MKL-1 OE
NC+Erastin
MKL-1 OE+Erastin

Figure S4

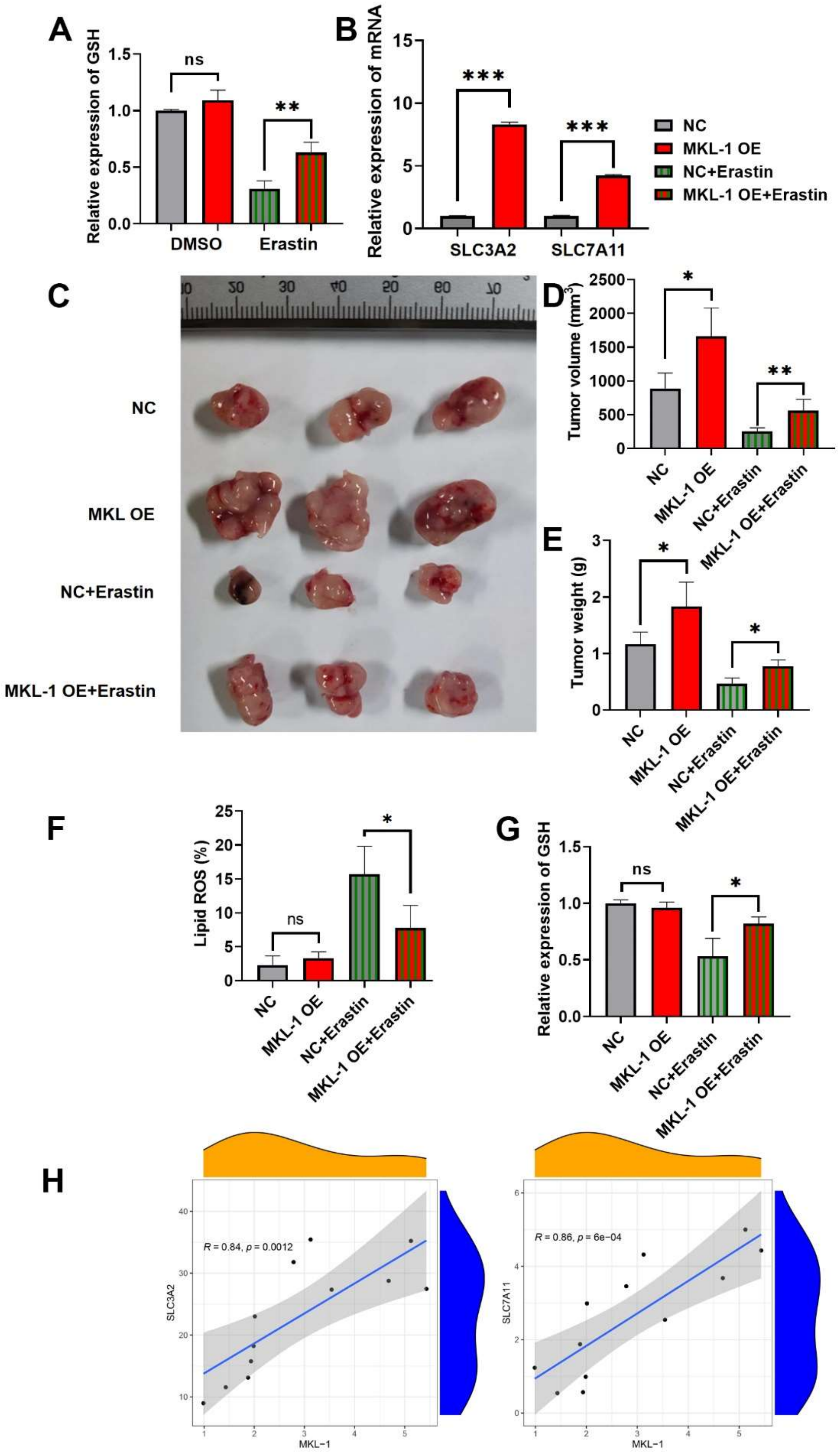

A
Relative expression of GSH
1.5
1.0
0.5
0.0
ns
**
DMSO
Erastin
B
Relative expression of mRNA
15
10
5
0
***
***
SLC3A2
SLC7A11
NC
MKL-1 OE
NC+Erastin
MKL-1 OE+Erastin
C
NC
MKL OE
NC+Erastin
MKL-1 OE+Erastin
D
Tumor volume (mm3)
2500
2000
1500
1000
500
0
*
**
NC
MKL-1 OE
NC+Erastin
MKL-1 OE+Erastin
E
Tumor weight (g)
3
2
1
0
*
*
NC
MKL-1 OE
NC+Erastin
MKL-1 OE+Erastin
F
Lipid ROS (%)
25
20
15
10
5
0
ns
*
NC
MKL-1 OE
NC+Erastin
MKL-1 OE+Erastin
G
Relative expression of GSH
1.5
1.0
0.5
0.0
ns
*
NC
MKL-1 OE
NC+Erastin
MKL-1 OE+Erastin
H
R = 0.84, p = 0.0012
SLC3A2
MKL-1
R = 0.86, p = 6e-04
SLC7A11
MKL-1

Figure S5

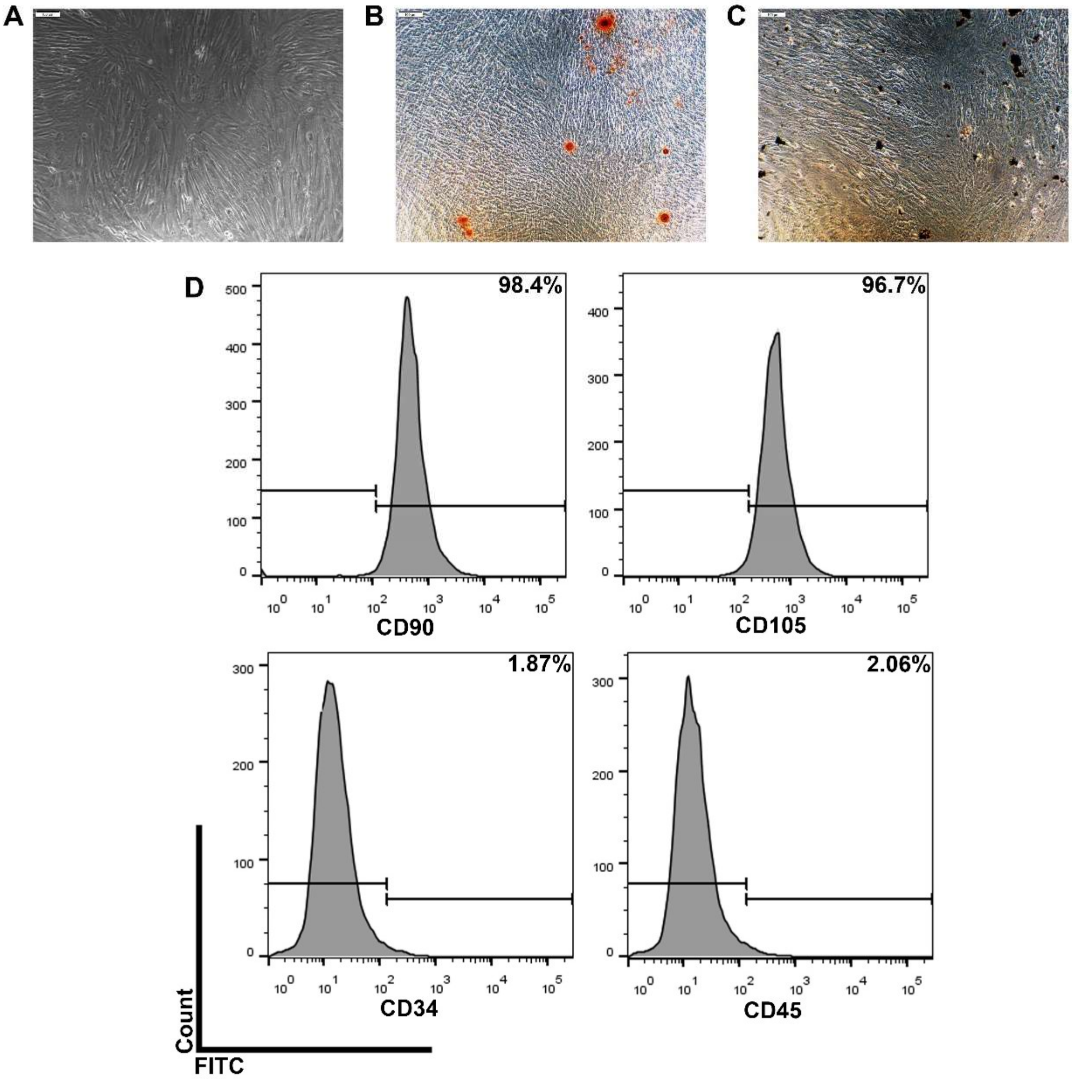

Figure S6

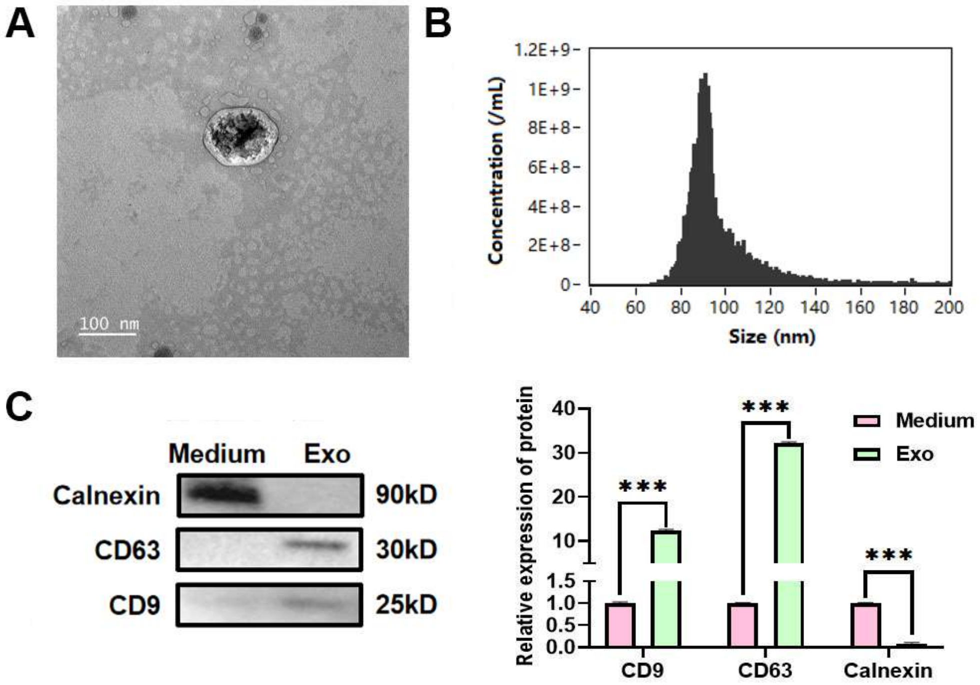
A
100 nm
B
Concentration (/mL)
1.2E+9
1E+9
8E+8
6E+8
4E+8
2E+8
0
40 60 80 100 120 140 160 180 200
Size (nm)
C
Medium Exo
Calnexin 90kD
CD63 30kD
CD9 25kD
Relative expression of protein
40
30
20
10
1.5
1.0
0.5
0.0
***
Medium
Exo
CD9 CD63 Calnexin

Figure S7

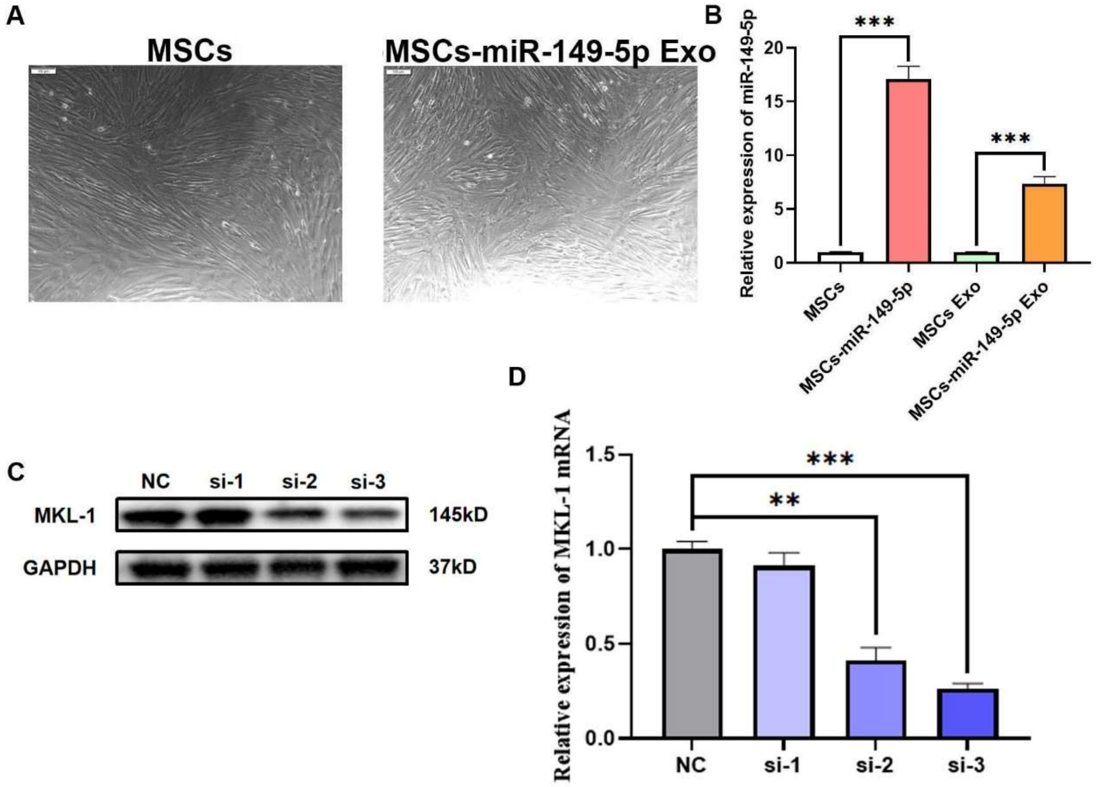
A
MSCs
MSCs-miR-149-5p Exo
B
Relative expression of miR-149-5p
***
***
20
15
10
5
0
MSCs
MSCs-miR-149-5p
MSCs Exo
MSCs-miR-149-5p Exo
C
NC
si-1
si-2
si-3
MKL-1
145kD
GAPDH
37kD
D
Relative expression of MKL-1 mRNA
***
**
1.5
1.0
0.5
0.0
NC
si-1
si-2
si-3

Figure S8

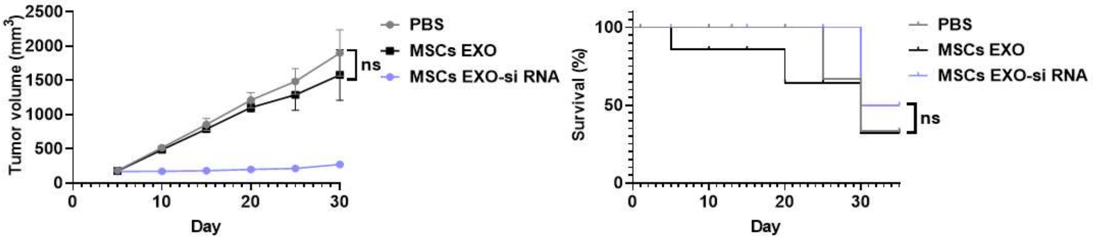
Tumor volume (mm3)
2500
2000
1500
1000
500
0
0
10
20
30
Day
ns
PBS
MSCs EXO
MSCs EXO-si RNA
Survival (%)
100
50
0
0
10
20
30
Day
ns
PBS
MSCs EXO
MSCs EXO-si RNA

Figure legends

Figure S1. Construction of ferroptosis stress model. A. CCK-8 assay was used to detect cell viability with different concentrations of ferroptosis inducer Erastin. B. CCK-8 assay was used to detect cell viability with different concentrations of ferroptosis inducer Erastin and ferroptosis inhibitor Fer-1. C. After the addition of ferroptosis inducer Erastin and ferroptosis inhibitor Fer-1, the Lipid ROS level of cells was detected by C11 BODIPY 581/591 probe and FCM. D. After the addition of ferroptosis inducer Erastin and ferroptosis inhibitor Fer-1, the level of the membrane lipid peroxidation product MDA was detected by the MDA lipid oxidation level detection kit. E. Typical images of mitochondria in MGC-803 and HGC-27 cells were observed by transmission electron microscopy at 80KV. The t-test was used to determine the statistical significance for both groups of analysis. The CCK-8 assay, Lipid ROS assay and MDA assay results represented the average values obtained from three independent experiments. The results presented were Mean ± SD. * $p < 0.05$, ** $p < 0.01$, *** $p < 0.001$, ns = $p > 0.05$.

Figure S2. Knockdown efficiency of MKL-1. A. The protein expression of MKL-1 in cells was detected by Western Blot. B. Image J software was used for the grayscale analysis of Western Blot results. C. The mRNA expression of MKL-1 was detected by qRT-PCR. D. The protein expression of MKL-1 in human gastric cancer cell lines AGS, MKN-45, HGC-27 and SGC-7901 were detected by Western Blot. The t-test was used to determine the statistical significance for both groups of analysis. The Western blot and qRT-PCR results represented the average values obtained from three independent

experiments. The results shown are Mean ± SD. ** $p < 0.01$.

Figure S3. Overexpression of MKL-1 promoted cystine uptake through System Xc-. A. Cystine (U-$^{13}C^{6}$, U-$^{15}N^{2}$) fed cells were used for analysis and GSH biosynthesis pathway by LC/MS. B. The isotope tracks the metabolism of cystine in the cell. The t-test was used to determine the statistical significance for both groups of analysis. The results shown are Mean ± SD. * $p < 0.05$, ** $p < 0.01$, *** $p < 0.001$, ns = $p > 0.05$.

Figure S4. Overexpression of MKL-1 reduces ferroptosis induced by Erastin in both in vitro and in vivo. A. The level of GSH in cells was detected by GSH detection kit. B. The mRNA expression of SLC3A2 and SLC7A11 in cells was detected by qRT-PCR. C. Typical images of the subcutaneous tumor tissue of nude mice after Erastin treatment (n = 3-5 tumors/group). D. The mass of subcutaneous tumor tissue in nude mice after Erastin treatment. E. The volume of subcutaneous tumor tissue in nude mice after Erastin treatment. F. The level of Lipid ROS in subcutaneous tumor tissue of nude mice was detected by C11 BODIPY 581/591 probe and FCM. G. The level of GSH in subcutaneous tumor tissue of nude mice was detected by GSH detection kit. H. Pearson correlation analysis of SLC3A2, SLC7A11 and MKL-1 mRNA expression in subcutaneous tumor tissue of nude mice. The t-test was used to determine the statistical significance for both groups of analysis. The GSH assay and qRT-PCR results represented the average values obtained from three independent experiments. The results shown are Mean ± SD. * $p < 0.05$, ** $p < 0.01$, *** $p < 0.001$, ns = $p > 0.05$.

Figure S5. Identification of MSCs. A. Typical images of MSCs cells under an inverted microscope. B. Typical images of alizarin red staining after 14 days of osteogenic

induction. C. Typical images of oil red O staining after 14 days of adipogenic induction. D. The expression of MSCs markers CD90, CD105, CD34 and CD45 was detected by FCM. The FCM result represented the average values obtained from three independent experiments. The results presented were Mean ± SD.

Figure S6. Extraction and identification of MSCs exosmoes. A. Typical images of exosomes observed by transmission electron microscopy at a voltage of 80KV. B. NTA analysis of exosome particle size. C. Western Blot detection of CD9, CD63 and Calnexin in MSCs supernatant and MSCs Exo Expression. D. Image J software for grayscale analysis of Western Blot results. The t-test was used to determine the statistical significance for both groups of analysis. The Western blot results represented the average values obtained from three independent experiments. The results presented were Mean ± SD in three independent trials. The results presented were Mean ± SD. *** $p < 0.001$.

Figure S7. MSCs Exo as a carrier to insert miR-149-5p and exogenous MKL-1 siRNA. A. Typical images of MSCs after lentivirus infection. B. The expression of miR-149-5p in cells and its Exo was detected by qRT-PCR. C. After transfection of MKL-1 siRNA exosome treatment 72 hours, the protein expression of MKL-1 in cells was detected by Western Blot. D. After transfection of MKL-1 siRNA exosome treatment, the mRNA expression of MKL-1 in cells was detected by qRT-PCR. The t-test was used to determine the statistical significance for both groups of analysis. The qRT-PCR result represented the average values obtained from three independent experiments. The results presented were Mean ± SD. ** $p < 0.01$, *** $p < 0.001$.

Figure S8. Therapeutic impact of exosomes on xenograft tumor models in nude mice. A. The volume of subcutaneous tumors formed in nude mice (n = 4-8 tumors/group). Multiple t-tests (followed by post-hoc test Holm-Sidak) was used to determine the statistical significance against control group. B. The Kaplan-Meier survival curve of nude mice (n = 10 tumors/group). Log-rank was used to determine the statistical significance. ns = $p > 0.05$.
